# Genetic Structure of the *Liriope muscari* Polyploid Complex and the Possibility of Its Genetic Disturbance in Japan

**DOI:** 10.3390/plants11223015

**Published:** 2022-11-08

**Authors:** Keita Watanabe, Makoto Yaneshita, Tetsuo Denda, Masatsugu Yokota, Shun K. Hirota, Yoshihisa Suyama, Yoshihiko Tsumura

**Affiliations:** 1 Graduate School of Life and Environmental Science, University of Tsukuba, Tsukuba 305-8572, Japan; 2 Environment Research Section Urban Engineering Research Department, Taisei Corporation, Yokohama 245-0051, Japan; 3 Laboratory of Ecology and Systematics, Faculty of Science, University of the Ryukyus, Nishihara 903-0213, Japan; 4 Botanical Gardens, Osaka Metropolitan University, Katano 576-0004, Japan; 5 Field Science Center, Graduate School of Agricultural Science, Tohoku University, Osaki 989-6711, Japan; 6 Faculty of Life and Environmental Sciences, University of Tsukuba, Tsukuba 305-8786, Japan

**Keywords:** polyploidy, greening, human disturbance, MIG-seq, conservation

## Abstract

Anthropogenic activities, such as the movement of plants through greening, can result in genetic disturbance that can interfere with local adaptation in wild populations. Although research is underway to prevent genetic disturbance associated with greening, genetic disturbance of intraspecific polyploidy, which is estimated to be present in 24% of vascular plants, has not been well studied. *Liriope muscari* is a polyploid complex with known diploid (2n = 36), tetraploid (2n = 72), and hexaploid (2n = 108) forms. The plants of this species tolerate dry and hot conditions and are therefore frequently used for greening and gardening. However, the distribution of this polyploid in Japan, its genetic structure, and genetic disturbance are not known. In this study, we investigated the polyploidy distribution and genetic structure in naturally distributed *L. muscari* in Japan using chloroplast DNA (cpDNA) haplotypes and nuclear DNA (nDNA). Commercially produced individuals were also studied and compared with natural populations to assess any genetic disturbance of the ploidy complex in this species. Chromosome counts, cpDNA, and nDNA results showed three genetically and cytologically distinct groups in Japan: first, a tetraploid group in mainland Japan; second, a hexaploid group in the Ryukyu Islands; and third, a diploid and tetraploid group in the Ryukyu Islands. Significant isolation by distance was also detected within the three groups (*p* = 0.001). Genetic disturbance due to greening and gardening should be avoided among the three groups. Genetic disturbance can be reduced by using individuals derived from natural populations that are close to the sites used for greening and gardening. For commercially produced individuals, genetic disturbance is unlikely in the Kanto region, an area of high usage, while genetic disturbance is thought possible in the Ryukyu Islands.

## 1. Introduction

The genetic structure of a population is formed by various mechanisms including the interaction of gene flow, genetic drift, and natural selection [1]. The genetic structure of populations may include adaptations to the local environment [2], which may be the starting point for expansion into different environments and speciation. Such genetic structure is important for the maintenance of genetic diversity and biodiversity.

Anthropogenic activity, such as plant transfer through greening, can result in genetic disturbance, which can affect a natural population by disrupting its genetic structure and disturbing any adaptation to local conditions [3]. Several studies have been conducted to prevent genetic disturbance caused by revegetation [4,5,6,7].

Polyploidy is common among plants; approximately 24% of vascular plants are polyploid [8]. Polyploidization may be harmful in the short term by causing karyotype instability and reducing opportunities for propagation, etc., but in the long term it is thought to play an important role in the creation of diversity and species differentiation. It can do this by reducing the manifestation of deleterious genes that can result from the redundancy of multiple genomes, enabling sexual differentiation and adaptation to different environments [9,10,11]. In plants with intraspecific polyploidy, each polyploid with its distinct characteristics is sometimes geographically limited to a specific habitat due to changes in ecological niches and reproductive isolation [12,13,14]. Despite the importance of polyploid plants in evolutionary processes, few studies on genetic disturbances in polyploid plants exist in the literature [15,16].

*Liriope muscari* (Decne.) Bailey is an evergreen perennial plant found in Mainland China, Taiwan, South Korea, and Japan. It is a polyploid species comprising three forms: diploid (2n = 36), tetraploid (2n = 72), and hexaploid (2n = 108) [17,18,19,20,21]. *L. muscari* is a useful plant with many applications and has been used as a herbal medicine since ancient times [22]. It is often used for greening because of its tolerance to drought and high temperature, and its high phytoremediation effect on Zn, Cu, Pb, Ni, and Cd in planted soil has been reported [23]. As of 2021, approximately 420,900 plants had been produced in Japan [24]. It is propagated by means of seeds from its hermaphrodite flowers. Its pollinators are not well documented, but visits by *Episyrphus* and *Parnara* are known (Watanabe pers. obs). Seeds are thought to be spread by birds [25], and the raccoon dog *Nyctereutes procyonoides viverrinus* is known to feed on the seed [26]. As mentioned above, *L. muscari*, which is heavily used for greening and gardening, is likely to be subject to genetic disturbance. However, the distribution of the polyploids in Japan, its genetic structure, and genetic disturbance are not known.

Here, we report our investigations into the distribution of polyploidy and the genetic structure of naturally distributed *L. muscari* in Japan using chloroplast DNA (cpDNA) haplotypes and nuclear DNA (nDNA). We also investigate commercially produced individuals to compare with the natural populations and discuss the genetic disturbance of the polyploidy complex in this species.

## 2. Results

### 2.1. Polyploidy Level

**Figure 1:**
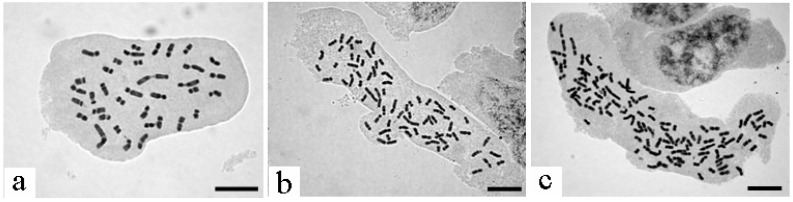
Chromosomes at mitotic metaphase of three polyploidy levels of *L. muscar*i. (**a**): diploid (2n = 36, sample name: wk179), (**b**): tetraploid (2n = 72, sample name: tm179), (**c**): hexaploid (2n = 108, sample name: wk006). Scale bars are 5 µm.

**Figure 2:**
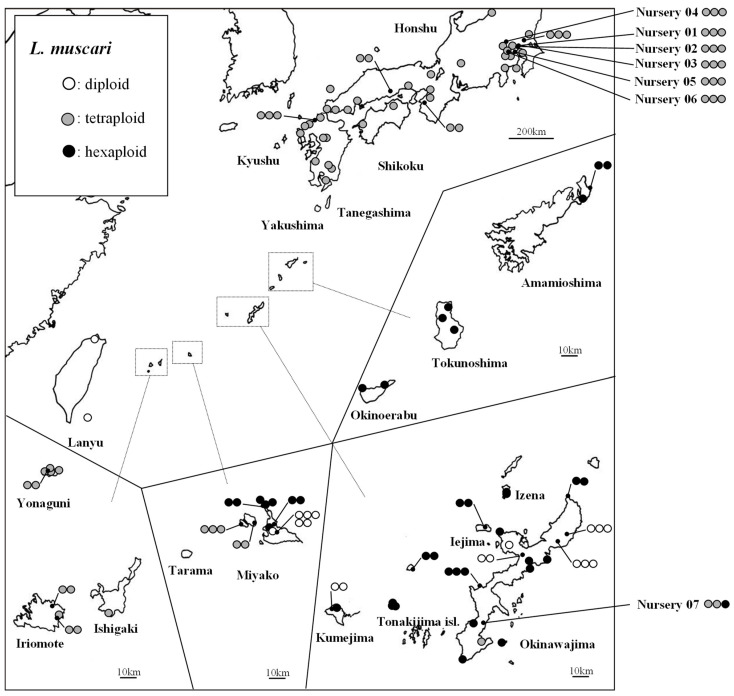
Geographic distributions of polyploidy levels observed in *L. muscari*.

The polyploidy level of 116 individuals at 86 sites were identified as follows (Table 1, Figure 1): 18 were diploid (8 sites), 61 were tetraploid (49 sites), and 37 were hexaploid (29 sites) (Table 1, Figure 1). We estimated the chromosome number using multiple individuals at 21 sites. The diploid individuals were found sporadically in the Ryukyu Islands and Taiwan, the tetraploids were found in mainland Japan and the southern Ryukyu Islands, and the hexaploids were found only in the central Ryukyu Islands. All 18 commercial individuals of *L. muscari* obtained from six nurseries in the Kanto region were tetraploid. Of the three individuals obtained from the nursery on the main island of Okinawa, two were tetraploid and one was hexaploid (Figure 2).

### 2.2. Chloroplast DNA Haplotypes

**Figure 3:**
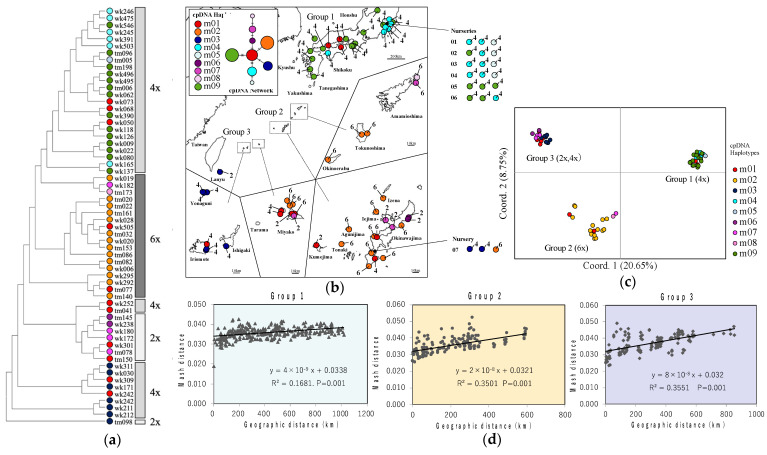
Geographical genetic structure of *L. muscari* using cpDNA and nDNA: (**a**) Neighbor-joining tree of *L. muscari*. Colored circles indicate differences in chloroplast DNA haplotypes (**b**). The numbers indicate the polyploidy level (2x: diploid 4x: tetraploid 6x: hexaploid). The color-coded areas indicate the three groups observed in the PCoA of the Mash distance (**c**). (**b**) Map of geographic distributions of haplotypes and polyploidy levels observed in *L. muscari*. Numbers indicate polyploidy level (2: diploid, 4: tetraploid, 6: hexaploid). TCS network of 9 cpDNA haplotypes (m01, m02, m03, m04, m05, m06, m07, m08, m09) observed in cytotypes (2x, 4x, 6x). Each line connecting two haplotypes represents a single mutation step. Circles indicate sample size. The color-coded areas indicate the distribution areas where individuals belonging to the three groups observed in the PCoA of the Mash distance (**c**). (**c**) PCoA analysis of Mash distance. The different colors of the plots indicate the chloroplast DNA haplotypes. The numbers on each axis indicate the percentage of variance. The color-coded areas represent plots of individuals belonging to the three visually recognized groups. (**d**) Correlation between Mash distance and geographic distance for the three groups observed in the PCoA of the Mash distance.

By combining the sequence data of four cpDNA regions that were analyzed, we identified nine haplotypes (m01, m02, m03, m04, m05, m06, m07, m08, and m09). The nucleotide substitutions and indels are shown in Table S1. The haplotype diversity (h) and nucleotide diversity (π) of *L. muscari* are shown in Table 2. TCS haplotype network based on a sequence with four regions combined showed that the central haplotype was m01. Haplotypes m02, m03, and m09 were confirmed as independent haplotypes, each with a single mutation step. Systematic connections were indicated between m04 and m05 and between m06, m07, and m08. In terms of the relationship between the haplotype network and the polyploids, of the nine haplotypes, a different polyploidy level was confirmed to be present within the same haplotype in m01, m03, and m07 (Figure 3b).

### 2.3. Nuclear DNA

The Mantel test for Mash distance to SNP-based genetic distance showed a significant correlation between data sets (*p* = 0.001). SNP analysis finally called 93 SNPs with a genotyping rate of over 90%, and sequence coverage averages were above 30 for all individuals. Stacks parameters adjusted during SNP call settings were -m = 8, R0.85, -*n* = 1. The statistics obtained from stacks-2.60 are shown in Table 3.

In the neighbor-joining tree output from Mashtree, three groups related to chloroplast DNA haplotypes and polyploidy were recognized: Group 1 includes mainland Japan and is tetraploid with major chloroplast DNA haplotypes m04 and m09; Group 2 is distributed in the Ryukyu Islands and is hexaploid with the major cpDNA haplotype m02; and Group 3, also distributed in the Ryukyu Islands, is diploid and tetraploid, with the major cpDNA haplotypes m01 and m03 (Figure 3a,b). Group 3 was identified as several closely related clusters composed of diploid and tetraploid (Figure 3a). PCoA plots also distinguished three groups (Figure 3c). Nuclear and chloroplast DNA results were generally consistent, but some discrepancies were observed. Haplotype m01 was found in all three groups, and haplotype m07 was commonly found in groups 2 and 3 (Figure 3a). Mash distance and geographic distance in the three groups were significantly correlated (*p* = 0.001). In contrast to the trend of genetic diversity for cpDNA, a trend of increasing genetic diversity with increasing polyploidy level was observed for nDNA (Table 2 and Table 3).

### 2.4. Genetic Characteristics of Commercially Produced L. muscari

**Figure 4:**
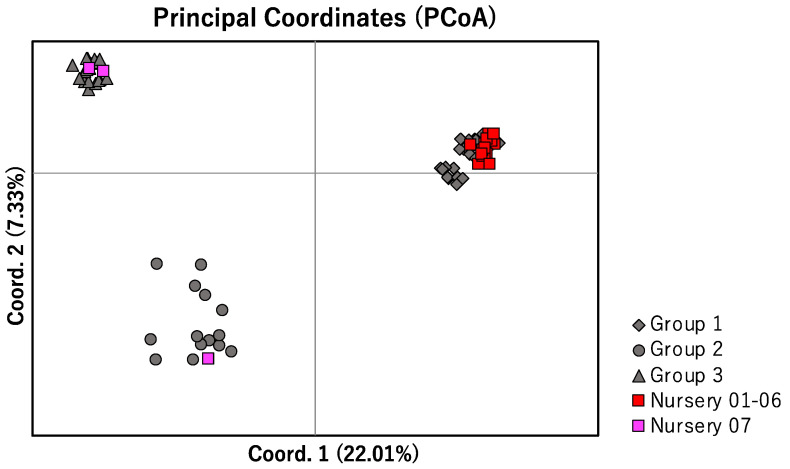
PCoA of wild individuals and cultivars by Mash distance. The different shapes of the gray symbols represent the three groups identified in wild individuals. The colored symbols indicate differences in nurseries. Nurseries 01–06 are in two adjacent prefectures in the Kanto region of Japan (Saitama and Tokyo). Nursery 07 is in Okinawa Prefecture, Japan.

The polyploidy, cpDNA haplotype, and nDNA characteristics of 18 *L. muscari* individuals obtained from nurseries 1–6 in the Kanto region were consistent with those naturally distributed around the nurseries. All were tetraploid, belonged to Group 1 identified by nDNA, and had cpDNA haplotypes m04, m05, and m09 (Figure 3b and Figure 4). Of the three individuals obtained from the nursery on Okinawa Island, one was hexaploid and had haplotype m02 and was included in Group 2. The remaining two individuals were tetraploid, had cpDNA haplotype m03, and belonged to Group 3. One individual in Group 2 matched the major type obtained from the Okinawa mainland, while two individuals in Group 3 had cpDNA haplotype m03, a type with a more southerly distribution (Figure 3b and Figure 4).

Genetic diversity of commercially produced *L. muscari* in nurseries in mainland Japan, with the main area of consumption near Tokyo, was comparable to the genetic diversity of Group 1 in mainland Japan in both cpDNA and nDNA (Table 2 and Table 3).

## 3. Discussion

### 3.1. Distribution of Polyploidy Complex

All *L. muscari* individuals sampled from mainland Japan were tetraploid in our study; but the Ryukyu Islands samples comprised a mix of three polyploids. However, the hexaploid *L. muscari* has been reported in Hiroshima, mainland Japan [17]; the hexaploid form is therefore probably distributed throughout mainland Japan but only at a low frequency. Previous studies have reported the diploid form in Zhejiang Province, China; tetraploid in Korea; and hexaploid in mainland Japan [17,19,20,27]. Combined with the present results, there may be considerable overlap in the distribution of polyploidy, with the diploid distributed from the Ryukyu Islands to Taiwan and Zhejiang Province, China; the tetraploid from mainland Japan to the Ryukyu Islands and Korea; and the hexaploid from mainland Japan to the Ryukyu Islands. In our study we were able to clarify the polyploidy distribution pattern roughly in Japan. However, due to the limited number of survey sites and individuals, the distribution of *L. muscari* polyploidy throughout its distribution range remains unknown in term of the frequency of polyploidy in each region.

Two closely related diploid–tetraploid pairs were identified in the neighbor-joining tree by Mash distance, suggesting that the tetraploids have multiple origins. The multiple origins of polyploidy has also been reported elsewhere [28,29]. The multiple origins of polyploidy may be one of the factors contributing to the geographic obscurity of the distribution of the *L. muscari* polyploid complex in the Ryukyu Islands.

### 3.2. Genetic Structure of L. muscari in Japan

The three groups clearly differed in nDNA were also mostly consistent with the results for polyploidy and cpDNA, but partial discrepancies were observed in cpDNA. Incomplete lineage sorting and chloroplast capture are the main causes of mismatching between nuclear and chloroplast DNA [30,31,32]. In our present study, both processes are also possible; but given the wide distribution for haplotype m01, which was one of the two chloroplast DNA haplotypes that did not match the nuclear DNA results, and the fact that it was found in all three groups with different polyploidy, strongly suggests a high probability of incomplete lineage sorting.

The diverse results for polyploidy, chloroplast DNA, and nuclear DNA among the three groups suggest that a genetic barrier to gene flow exists between these groups. In general, geographic isolation and climatic conditions are known to be barriers to gene flow [33,34,35]. The adjacent Groups 1 and 2 are separated by the Strait of Tokara, which is known to have a different flora due to geographic isolation [35]. While Group 1 is tetraploid, Group 2 is hexaploid, and such differences in chromosome number are also a barrier to gene flow [36]. The boundary between Group 1 and Group 2 distributions is unclear, probably due to low sampling density, but multiple barriers may also prevent gene flow.

It is difficult to explain the geographic and climatic barriers to gene flow between Groups 2 and 3, whose distributions overlap in the Ryukyu Islands. Since Group 2 is hexaploid and Group 3 is diploid and tetraploid, a barrier to gene flow due to reproductive isolation at different polyploidy levels can be inferred. Group 3 includes diploid and tetraploid, but within the wild population the ploidy level is fixed to either diploid or tetraploid. Fixation of the polyploidy level in each local population suggests that diploid and tetraploid may be exclusive. It may be possible that gene flow is restricted between polyploids within Group 3.

The relationship between Mash distance and geographic distance showed a significant correlation for all groups. Restricted interbreeding between geographically distant individuals results in IBD [37]. *L. muscari* is a common species at low elevations within the range of our collection of samples. The possibility of restricted gene flow between diploids and tetraploids in Group 3 should be noted, but, in any case, it suggests that geographic proximity is important for interbreeding within groups.

We found lower cpDNA diversity of hexaploid individuals in the Ryukyu Islands compared to other polyploidy levels (h = 0.399, π = 0.00030). If both haplotype diversity and nucleotide diversity are low, the hexaploid may have experienced a more recent origin or a more restrictive bottleneck compared to other polyploidy levels. In either case, the hexaploid in the Ryukyu Islands is likely dependent on limited genetic sources. On the other hand, the higher genetic diversity of hexaploids compared to diploids and tetraploids in nDNA may reflect the increased diversity associated with genome duplication.

### 3.3. Taxonomic Confusion

The genus *Liriope* exhibits a certain amount of taxonomic confusion. *L. tawadae* is characterized by large plant size, broad and long leaf blades, and large flowers and long flower stalks, and has been reported in the Ryukyu Islands [38]. Due to the lack of morphological information, the relationship between *L. tawadae* and the result of this study is unclear. Some cpDNA haplotypes of *L. muscari* are shared with those of *L. spicata* (Watanabe, unpublished data), suggesting past hybridization. The situation is further complicated because *L. spicata* is known to be diploid, tetraploid, and hexaploid [19,21,39]. Further taxonomic reexamination, including related species, is therefore needed.

### 3.4. Potential of Anthropogenic Disturbance and Countermeasures

The commercially produced *L. muscari* was abundant near Tokyo; however, there was no obvious risk of genetic disturbance evident from our study. All *L. muscari* produced in nurseries near Tokyo were confirmed to be tetraploid and genetically close to naturally distributed individuals in the neighborhood. On the other hand, there is a possibility of genetic disturbance of *L. muscari* in the Ryukyu Islands due to greening. Despite our limited number of samples, we observed that genetically distinct *L. muscari* were being sold together. In addition, individuals from genetically distinct groups that were sold together also differed in their polyploidy levels, with one of the three individuals studied being Group 2 hexaploid and two being Group 3 tetraploids. The mixing of different polyploidy levels can cause additional problems. It has been noted that orthotopic growth of different polyploidy levels causes the eradication of minor polyploidy levels [40]. In fact, the polyploidy levels were fixed in populations where chromosome counts of multiple individuals were examined in this study. It should also be noted that pentaploids can easily be obtained by artificially crossing tetraploids and hexaploids from the Ryukyu Islands (Watanabe, unpublished data).

An effective means of preventing anthropogenic genetic disturbances of *L. muscari* is to avoid contact between genetically distinct groups. By selecting *L. muscari* that belong to the same genetic group as the natural population surrounding the proposed greening site, and by using seed collected from a single group during seedling production, contact between genetically distinct groups can be reduced. Considering within groups, genetic distance between geographically close individuals is close, so using individuals derived from natural populations that are geographically close to the proposed greening site is expected to further reduce genetic disturbance. Group 3 suggests multiple origins of the tetraploid, and although the situation may be complex, it is expected that the supply of individuals with the same polyploidy level from a natural population near the proposed site at the time of greening will reduce genetic disturbance.

Determining a level of genetic disturbance based on genetic information remains difficult for greening officials. With *L. muscari* in Japan, it is possible to recognize groups that should avoid contact with each other based on their geographic distribution and polyploidy levels. Three groups in Japan should avoid being genetically disturbed. The first group is the tetraploid distributed in mainland Japan. There are also two groups in the Ryukyu Islands that have overlapping geographic boundaries but can be distinguished by polyploidy level. The second group is the hexaploid of the Ryukyu Islands. The third group is the diploid and tetraploid of the Ryukyu Islands. At present it is difficult to estimate the polyploidy level of *L. muscari* by morphological features, but accumulating such morphological information will be an effective way to test for correspondence with the polyploidy level in order to aid their recognition in the field.

## 4. Materials and Methods

### 4.1. Collection of Materials

We collected between one and five individuals from 86 sites (totaling 116 individuals) in the natural distribution area, ranging from Niigata Prefecture in Japan to Taiwan. A further 21 commercially produced individuals were also collected: three each from two nurseries in Tokyo, four nurseries in Saitama Prefecture, and one nursery in Okinawa Prefecture (Table 1).

### 4.2. Determination of Polyploid Level

For each of the 137 *L. muscari* individuals collected, we counted their chromosome number using the aceto-orcein squash method. The root tip meristems were placed in a 0.002 M 8-hydroxyquinoline solution and pretreated at room temperature for 4–5 h. Subsequently, they were left in acetic acid alcohol (3:1, anhydrous ethanol–glacial acetic acid) to harden for at least five hours at 4 °C. After hardening, they were disaggregated for approximately 40 s in a 60 °C disaggregation solution (2:1, 1N hydrochloric acid–45% acetic acid) and stained for 1 to 15 min in a 2% aceto-orcein solution, and then squashed on a glass slide. The number of chromosomes was counted in somatic cells at metaphase.

### 4.3. Chloroplast DNA Analysis

The total DNA was extracted following the CTAB method of Doyle and Doyle (1987) after removing polysaccharides using the method of Setoguchi and Ohba (1995) [41,42].

Using four pairs of primers developed by Taberlet, (1991), Denda and Yokota (2003), Nakamura et al. (2006), Liston and Kadereit (1995) [43,44,45,46], polymerase chain reaction (PCR) amplification was conducted for the following intron and intergenic spacers and regions: *trn*K 5′ intron: (5′-CTCAACGGTAGAGTACTCG-3′, 5′-CCAAAAACTTCCACAGGTTCG-3′), *trn*T-*trn*L: (5′-GCGATGCTCTAACCTCTGAG-3′, 5′-TAGCGTCTACCGATTTCGCC-3′), *trn*L-*trn*F: (5′-ATTTGAACTGGTGACACGAG-3′, 5′-ATTTGAACTGGTGACACGAG-3′), and *atp*B-*rbc*L: (5′-ACTTAGAGGAGCTCCCGTGTCAATC-3′, 5′-GAGTTACTCGGAATGCTGCC-3′) intergenic regions. PCR amplification was performed using a PCR Thermal cycler SP (Takara), and base sequence determination was performed using a CEQ 8800 capillary DNA sequencer (Beckman Coulter). The base sequence obtained in this manner was aligned using the default parameters of the ClustalW program implemented in the MEGA X software [47]. The chloroplast DNA haplotypes were detected based on the arrangement of 3021 bases for the four combined domains. In addition, using DnaSP version 6.12.03 [48], the haplotypic diversity (h) and nucleotide diversity (π) were calculated for each polyploid and each region in which differences in polyploid distribution had been determined [49]. A parsimony haplotype network diagram of chloroplast DNA was created using the PopART 1.7 TCS network based on the data set in which the base sequences for the *trn*K intron, the *trn*T-L, *trn*L-F and *atp*B-*rbc*L intergenic regions [50], and the *mat*K gene region were combined. Insertion–deletion (INDEL) mutations were excluded from analysis on the TCS network, so a dataset was created with INDELs replaced with base substitutions. We excluded from the analysis all repeated insertion–deletion of sequences for which the homology of the mutations was unclear.

### 4.4. Nuclear DNA Analysis

Nuclear DNA was investigated by sequencing with MIG-seq analysis [51,52], which creates a reduced library of genomes for samples with known chromosome numbers and chloroplast DNA haplotypes. The region flanked by SSRs was PCR amplified using 8 primers in the first PCR, and the resulting amplicons were indexed in a second PCR. The indexed DNA library was sequenced using the MiSeq Reagent Kit v3 (150-cycle) (Illumina, San Diego, CA, USA). The obtained reads were filtered using Trimmomatic 0.39 [53]. Adapter sequences were removed using default settings and short reads were removed (MINLEN:79). Low quality reads were then removed and trimmed (SLIDINGWINDOW:4:15, CROP:79). To investigate genetic distances between individuals, we used Mashtree [54] to create a neighbor-joining tree based on Mash distance between individuals. Mashtree parameters were k-mer length 21 (--kmerlength 21), sketch k-mer count was set to 30,000 (--sketchsize 30,000), and k-mers with a count of less than 2 were excluded from the analysis (--mindepth 2).

The genetic analysis of polyploid data involves certain challenges. The main one is the difficulty in estimating allele frequencies of the polyploid. Many existing analysis methods require allele doses, for example, hundreds of coverages to recover tetraploid alleles with 90% confidence [55]. It is difficult to obtain this amount of information by normal greening. k-mer analysis using the MinHash method does not require allele dosage, thus alleviating the difficulties of polyploid analysis [56]. However, Mashtree-derived Mash distances provide information equivalent to genetic distances. They are obtained by evaluating the similarity of reads resolved into k-mers. To assess the similarity between individuals, Principal Coordinate Analysis (PCoA) using Mash distance was performed in GenAlex 6.502. Mantel tests were performed on GenAlex 6.502 for Mash distance and geographic distance to estimate isolation by distance (IBD) between the groups recognized by PCoA (Tables S2–S4). However, such data should be treated with caution when evaluating between different polyploidy levels using Mash distances, as they can be subject to bias [56]. To assess the plausibility of comparisons between different polyploidy levels using the Mash distance, a Mantel test was performed on the genetic distance obtained from single nucleotide polymorphisms (SNP) and the Mash distance (Table S5). The Mantel test was performed using GenAlex 6.502, and the SNP call was made using the denovo_map.pl pipeline from stacks-2.60 [57]. The parameters for estimating the genetic diversity of *L. muscari* were also obtained with stacks-2.60. Stacks is an analytical protocol for a diploid model, which is usually difficult to apply to a polyploid. Although several informative alleles are lost, stacks can be used to analyze polyploidy by treating them as diploid by linking copies derived from polyploidy [58].

## 5. Conclusions

In Japan, there are three groups of natural populations of *L. muscari* recognized by polyploidy, cpDNA, and nDNA: the first is the mainland tetraploid group; the second is the Ryukyu Islands hexaploid group; and the third is the Ryukyu Islands diploid and tetraploid group. For the reduction of potential risks regarding the destruction of the local adaptation of natural individuals around the greening area and for the establishment of the planted individuals, genetic disturbance associated with greening between these three groups must be avoided. In the Kanto region near large cities, the possibility of genetic disturbance due to greening is low because the cultivated products and the surrounding natural populations belong to the same group. On the other hand, in the Ryukyu Islands, individuals belonging to different groups were being sold in the same nursery, suggesting the possibility of genetic disturbance between groups due to greening. Within the three groups, distinct IBD could be identified in nDNA. Using individuals derived from natural populations that are geographically close to the proposed greening site is expected to further reduce genetic disturbance.

## Figures and Tables

**Table 1:** Plant materials and localities of samples used in this study. Na: sample names, 2n: chromosome numbers, Hap: cpDNA haplotype names.

Locality	Na	2n	Hap	Accession Numbers				
				cpDNA				nDNA
				*trn*K 5′ Intron	*trn*T-*trn*L	*trn*L-*trn*F	*atp*B-*rbc*L	
Natural distribution area								
Mainland Japan and Ryukyu Islands								
Kakudayama, Niigata City, Niigata Pref.	wk390	72	m09	LC730908	LC731009	LC731090	LC731171	DRR412610
Mt. Tsukuba, Tsukuba City, Ibaraki Pref.	tm096	72	m09	LC730909	LC731010	LC731091	LC731172	DRR412611
	tm001	72						
	tm179	72						
Mt. Mayumi, Hitachiota City, Ibaraki Pref.	wk270	72						
Arakawa river, Nagatoro Town, Saitama Pref.	tm005	72	m05	LC730910	LC731011	LC731092	LC731173	DRR412612
Kamitanadare, Kisai Town, Saitama Pref.	wk018	72						
Sendabori, Matsudo City, Chiba Pref.	wk246	72	m04	LC730911	LC731012	LC731093	LC731174	DRR412613
Horiuchinai, Ichikawa City, Chiba Pref.	wk503	72	m04	LC730912	LC731013	LC731094	LC731175	DRR412614
Mogusa, Tama City, Tokyo Pref.	tm003	72						
Hane, Hamura City, Tokyo Pref.	wk546	72	m09	LC730913	LC731014	LC731095	LC731176	DRR412615
Motohachioji, Hachioji City, Tokyo Pref.	wk475	72	m04	LC730914	LC731015	LC731096	LC731177	DRR412616
Horiuchi, Hayama Town, Kanagawa Pref.	wk391	72	m04	LC730915	LC731016	LC731097	LC731178	DRR412617
Hakone, Hakone Town, Kanagawa Pref.	wk245	72	m04	LC730916	LC731017	LC731098	LC731179	DRR412618
Siokawa, Kani City, Gifu Pref.	tm198	72	m09	LC730917	LC731018	LC731099	LC731180	DRR412619
Imodani, Hashimoto City, Wakayama Pref.	tm006	72	m09	LC730918	LC731019	LC731100	LC731181	DRR412620
Hasemiya, Kimino Town, Wakayama Pref.	wk495	72	m09	LC730919	LC731020	LC731101	LC731182	DRR412621
	wk496	72	m09	LC730920	LC731021	LC731102	LC731183	DRR412622
Mt. Kurama, Sakyou Ward, Kyoto Pref.	wk278	72						
Mt. Takao, Kashiwara City, Osaka Pref.	tm008	72						
Higashiune, Akou City, Hyogo Pref.	wk062	72	m09	LC730921	LC731022	LC731103	LC731184	DRR412623
Asagoe, Okayama City, Okayama Pref.	wk067	72						
	wk068	72	m01	LC730922	LC731023	LC731104	LC731185	DRR412624
Mt. Ogonzan, Hiroshima City, Hiroshima Pref.	wk073	72	m01	LC730923	LC731024	LC731105	LC731186	DRR412625
Chuocho, Hikari City, Yamaguchi Pref.	wk080	72	m09	LC730924	LC731025	LC731106	LC731187	DRR412626
Tyuzankei, Shimonoseki City, Yamaguchi Pref.	tm012	72						
Nagaonohana, Hagi City, Yamaguchi Pref.	wk022	72	m09	LC730925	LC731026	LC731107	LC731188	DRR412627
Onoyama, Sanyo Onoda City, Yamaguchi Pref.	wk024	72						
Kishinoue, Mannou Town, Kagawa Pref.	wk050	72	m01	LC730926	LC731027	LC731108	LC731189	DRR412628
Sugeta, Ohzu City, Ehime Pref.	wk165	72	m04	LC730927	LC731028	LC731109	LC731190	DRR412629
Nagahama seashore, Hukuoka City, Fukuoka Pref.	wk009	72	m09	LC730928	LC731029	LC731110	LC731191	DRR412630
	wk010	72						
	wk011	72						
Mt. Ihara, Maebaru City, Fukuoka Pref.	wk013	72						
Senbutsudo, Kokura City, Fukuoka Pref.	tm014	72						
Mt. Kagamiyama, Karatsu City, Saga Pref.	wk015	72						
Hae, Tano Town, Miyazaki Pref.	wk255	72						
Okutsu, Kobayashi City, Miyazaki Pref.	wk256	72						
Tatara, Ozu City, Kumamoto Pref.	wk134	72						
Ino, Kikuchi City, Kumamoto Pref.	wk137	72	m09	LC730929	LC731030	LC731111	LC731192	DRR412631
Mt. Tokozan, Izumi City, Kagoshima Pref.	wk126	72	m09	LC730930	LC731031	LC731112	LC731193	DRR412632
Hatimanjinja, Kanoya City, Kagoshima Pref.	wk118	72	m09	LC730931	LC731032	LC731113	LC731194	DRR412633
Tomori, Amami City, Kagoshima Pref.	tm169	108						
	tm173	108	m08	LC730932	LC731033	LC731114	LC731195	DRR412634
Oazasetsuta, Amami City, Kagoshima Pref.	wk182	108	m07	LC730933	LC731034	LC731115	LC731196	DRR412635
Mt. Amagi, Amagi Town, Kagoshima Pref.	tm022	108	m02	LC730934	LC731035	LC731116	LC731197	DRR412636
Syoda, Tokunoshima Town, Kagoshima Pref.	tm019	108	m02	LC730935	LC731036	LC731117	LC731198	DRR412637
San, Tokunoshima Town, Kagoshima Pref.	wk548	108						
Kibiru, Wadomari Town, Kagoshima Pref.	tm021	108						
Taminazaki, China Town, Kagoshima Pref.	tm020	108	m02	LC730936	LC731037	LC731118	LC731199	DRR412638
Rikugidara, Izena Vil., Okinawa Pref.	tm168	108						
Mt. Chizin, Izena Vil., Okinawa Pref.	tm086	108	m02	LC730937	LC731038	LC731119	LC731200	DRR412639
Mt. Gusuku, Ie Vil., Okinawa Pref.	wk028	108	m02	LC730938	LC731039	LC731120	LC731201	DRR412640
	wk029	108						
Cape Hedo, Kunigami Vil., Okinawa Pref.	tm038	108						
	tm144	108						
Uka river, Kunigami Vil., Okinawa Pref.	tm037	36						
	tm145	36	m06	LC730939	LC731040	LC731121	LC731202	DRR412641
	tm146	36						
Haramata river, Higashi Vil., Okinawa Pref.	wk236	36						
	wk237	36						
	wk238	36	m06	LC730940	LC731041	LC731122	LC731203	DRR412642
Bise, Motobu Town, Okinawa Pref.	tm149	108						
Mt. Awa, Motobu Town, Okinawa Pref.	wk172	36	m07	LC730941	LC731042	LC731123	LC731204	DRR412643
Kushi, Nago City, Okinawa Pref.	tm029	108						
Henoko, Nago City, Okinawa Pref.	tm148	108						
Mt. Nago, Nago City, Okinawa Pref.	wk179	36						
	wk180	36	m07	LC730942	LC731043	LC731124	LC731205	DRR412644
Cape Maeda, Onna Vil, Okinawa Pref.	tm031	108						
	tm032	108	m02	LC730943	LC731044	LC731125	LC731206	DRR412645
	wk505	108	m01	LC730944	LC731045	LC731126	LC731207	DRR412646
Ojana, Ginowan City, Okinawa Pref.	wk006	108	m02	LC730945	LC731046	LC731127	LC731208	DRR412647
Sashikisinzato, Nanjo City, Okinawa Pref.	wk242	72	m01	LC730946	LC731047	LC731128	LC731209	DRR412648
Cape Kyan, Itoman City, Okinawa Pref.	wk020	108	m02	LC730947	LC731048	LC731129	LC731210	DRR412649
Ugu seashore, Aguni Vil., Okinawa Pref.	tm152	108						
	tm153	108	m02	LC730948	LC731049	LC731130	LC731211	DRR412650
West side of Gityuyama, Tonaki Vil., Okinawa Pref.	tm092	108						
Iri, Tonaki Vil., Okinawa Pref.	tm095	108						
Womozaki, Tonaki Vil., Okinawa Pref.	tm161	108	m02	LC730949	LC731050	LC731131	LC731212	DRR412651
Mt. Ara, Kumejima Town, Okinawa Pref.	tm150	36	m01	LC730950	LC731051	LC731132	LC731213	DRR412652
	wk368	36						
Hiyajo, Kumejima Town, Okinawa Pref.	tm151	108						
Ishiki seashore, Nanjo City, Okinawa Pref.	tm082	108	m02	LC730951	LC731052	LC731133	LC731214	DRR412653
Hiraraogami, Miyakojima City, Okinawa Pref.	wk295	108	m02	LC730952	LC731053	LC731134	LC731215	DRR412654
Maesato, Miyakojima City, Okinawa Pref.	tm140	108	m02	LC730953	LC731054	LC731135	LC731216	DRR412655
Nishihennazaki, Miyakojima City, Okinawa Pref.	tm142	108						
	wk297	108						
Setozaki, Miyakojima City, Okinawa Pref.	wk292	108	m02	LC730954	LC731055	LC731136	LC731217	DRR412656
Onosanrin, Miyakojima City, Okinawa Pref.	tm077	108	m01	LC730955	LC731056	LC731137	LC731218	DRR412657
	wk291	108						
Otakikoen, Miyakojima City, Okinawa Pref.	tm076	36						
	tm078	36	m07	LC730956	LC731057	LC731138	LC731219	DRR412658
	tm079	36						
	tm143	36						
	wk298	36						
Nobarudake, Miyakojima City, Okinawa Pref.	wk301	36	m01	LC730957	LC731058	LC731139	LC731220	DRR412659
Umarezatonoutaki, Miyakojima City, Okinawa Pref.	wk334	108						
Mt. Makiyama, Miyakojima City, Okinawa Pref.	tm111	72						
	wk289	72						
Kuninakautaki, Miyakojima City, Okinawa Pref.	wk252	72	m01	LC730958	LC731059	LC731140	LC731221	DRR412660
Toriike, Miyakojima City, Okinawa Pref.	tm041	72	m01	LC730959	LC731060	LC731141	LC731222	DRR412661
	tm044	72						
	tm134	72						
Misakiutaki, Ishigaki City, Okinawa Pref.	wk171	72	m03	LC730960	LC731061	LC731142	LC731223	DRR412662
Yutsun river, Taketomi Town, Okinawa Pref.	tm117	72						
	wk309	72	m01	LC730961	LC731062	LC731143	LC731224	DRR412663
Komi, Taketomi Town, Okinawa Pref.	wk311	72	m03	LC730962	LC731063	LC731144	LC731225	DRR412664
	wk312	72						
Aira river, Taketomi Town, Okinawa Pref.	wk030	72	m03	LC730963	LC731064	LC731145	LC731226	DRR412665
Thindahanata, Yonaguni Town, Okinawa Pref.	tm115	72						
Agarizaki, Yonaguni Town, Okinawa Pref.	wk215	72	m03	LC730964	LC731065	LC731146	LC731227	DRR412666
Mt. Kubura, Yonaguni Town, Okinawa Pref.	tm113	72						
	tm114	72						
Nama seashore, Yonaguni Town, Okinawa Pref.	wk211	72	m03	LC730965	LC731066	LC731147	LC731228	DRR412667
Yonaguni, Yonaguni Town, Okinawa Pref.	wk212	72	m03	LC730966	LC731067	LC731148	LC731229	DRR412668
Taiwan								
Chingching-tsaoyan, Lanyu, Taitung	tm098	36	m03	LC730967	LC731068	LC731149	LC731230	DRR412669
Nurseries								
Nursery 01, Kawaguchi City, Saitama Pref.	wk460	72	m05	LC730968	LC731069	LC731150	LC731231	DRR412670
	wk461	72	m04	LC730969	LC731070	LC731151	LC731232	DRR412671
	wk462	72	m04	LC730970	LC731071	LC731152	LC731233	DRR412672
Nursery 02, Kawaguchi City, Saitama Pref.	wk463	72	m09	LC730971	LC731072	LC731153	LC731234	DRR412673
	wk464	72	m04	LC730972	LC731073	LC731154	LC731235	DRR412674
	wk465	72	m05	LC730973	LC731074	LC731155	LC731236	DRR412675
Nursery 03, Kawaguchi City, Saitama Pref.	wk466	72	m05	LC730974	LC731075	LC731156	LC731237	DRR412676
	wk467	72	m04	LC730975	LC731076	LC731157	LC731238	DRR412677
	wk468	72	m04	LC730976	LC731077	LC731158	LC731239	DRR412678
Nursery 04, Yorii Town, Saitama Pref.	wk469	72	m04	LC730977	LC731078	LC731159	LC731240	DRR412679
	wk470	72	m04	LC730978	LC731079	LC731160	LC731241	DRR412680
	wk471	72	m05	LC730979	LC731080	LC731161	LC731242	DRR412681
Nursery 05, Musashimurayama City, Tokyo Pref.	wk500	72	m09	LC730980	LC731081	LC731162	LC731243	DRR412682
	wk501	72	m04	LC730981	LC731082	LC731163	LC731244	DRR412683
	wk502	72	m09	LC730982	LC731083	LC731164	LC731245	DRR412684
Nursery 06, Chohu City, Tokyo Pref.	wk472	72	m09	LC730983	LC731084	LC731165	LC731246	DRR412685
	wk473	72	m09	LC730984	LC731085	LC731166	LC731247	DRR412686
	wk474	72	m09	LC730985	LC731086	LC731167	LC731248	DRR412687
Nursery 07, Nishihara City, Okinawa Pref.	wk497	72	m03	LC730986	LC731087	LC731168	LC731249	DRR412688
	wk498	72	m03	LC730987	LC731088	LC731169	LC731250	DRR412689
	wk499	108	m02	LC730988	LC731089	LC731170	LC731251	DRR412690

**Table 2:** cpDNA haplotype diversity and nucleotide diversity observed in *L. muscari.* N: number of samples, NH: number of haplotypes, h: haplotype diversity, π: nucleotide diversity.

Groups	Polyploidy	N	NH	h	π
Natural distribution area					
Group 1: Mainland Japan	4x	24	3	0.583	0.00062
Group 2: Ryukyu Islands	6x	18	4	0.399	0.00030
Group 3: Ryukyu Islands	2x, 4x	18	4	0.739	0.00041
Group 3–1	2x	8	4	0.821	0.00044
Group 3–2	4x	10	2	0.533	0.00018
Nurseries					
Nursery 01–06: Mainland Japan	4x	18	2	0.471	0.00063
Nursery 07: Ryukyu Islands	4x	2	1	0.000	0.00000
Nursery 07: Ryukyu Islands	6x	1	1	-	-

**Table 3:** nDNA Genetic diversity of *L. muscari*. N: number of samples, He: genetic diversity, Ho: observed heterozygosity, *F*_IS_: inbreeding coefficient, π: nucleotide diversity.

Groups	Polyploidy	N	He	Ho	*F*_IS_	π
Natural distribution area						
Group 1: Mainland Japan	4x	24	0.00262	0.00283	−0.00052	0.00268
Group 2: Ryukyu Islands	6x	18	0.00377	0.00382	0.00061	0.00390
Group 3: Ryukyu Islands	2x, 4x	18	0.00348	0.00256	0.00374	0.00360
Group 3-1	2x	8	0.00178	0.00104	0.00294	0.00196
Group 3-2	4x	10	0.00254	0.00299	−0.00093	0.00262
Nurseries						
Nursery 01-06: Mainland Japan	4x	18	0.00254	0.00299	−0.00093	0.00262
Nursery 07: Ryukyu Islands	4x	2	0.00234	0.00371	−0.00018	0.00359
Nursery 07: Ryukyu Islands	6x	1	0.00301	0.00602	0	0.00602

## Data Availability

Sequence data have been submitted to the GenBank database (cpDNA: LC730908-LC730988, LC731009-LC731251, nDNA: DRR412610-DRR412690).

## Supplementary Materials

The following supporting information can be downloaded at: https://www.mdpi.com/article/10.3390/plants11223015/s1, Table S1: Nucleotide substitutions and indels observed in the *Liriope muscari*; Table S2: Geographic distance (below) and Mash distance (above) for Group 1; Table S3: Geographic distance (below) and Mash distance (above) for Group 2; Table S4: Geographic distance (below) and Mash distance (above) for Group 3; Table S5: Marsh distance (below) and SNP-based genetic distance (above) for all individuals.
