# Supplementary material for: Genetic Structure of the Liriope muscari Polyploid Complex and the Possibility of Its Genetic Disturbance in Japan
Source: Plants (Basel). 2022 Nov 8;11(22):3015. doi: 10.3390/plants11223015 (PMC9697476; doi:10.3390/plants11223015)
Supplement: Supplementary file 1 [file plants-11-03015-s001.zip › plants-1991501-supplementary.pdf]

Table S1. Nucleotide substitutions and indels observed in the *Liriope muscari.* Nucleotides identical to the consensus are indicated by *, and gaps are indicated by -.

**Species**	**Haplotype**	***trnK* 5' intron**				***trn*T-*trn*L**					***trn*L-*trn*F**		***atp*B-*rbc*L**	
	m01	C	C	C	A	C	ACTAA	A	T	T	A	G	G	G
	m02	*	*	*	*	*	*	*	*	*	G	*	*	*
	m03	*	*	*	*	*	*	*	*	*	*	*	A	*
	m04	T	*	*	*	*	*	*	*	C	*	*	*	*
L. muscari	m05	T	*	*	*	*	-----	*	*	C	*	*	*	*
	m06	*	*	*	*	*	*	*	*	*	*	*	*	T
	m07	*	*	*	*	G	*	*	*	*	*	*	*	T
	m08	*	*	*	*	G	*	*	*	*	*	A	*	T
	m09	*	T	A	*	*	*	*	*	*	*	*	*	*

Table S2. Geographic distance (below) and Mash distance (above) for Group 1. Geographic distance is the distance between GPS coordinates (m). Isolation by distance (IBD) was tested by the Mantel test of GenAlex 6.502.

	wk062	wk475	wk022	wk245	wk546	tm006	wk391	wk080	wk073	wk009	wk503	wk126	tm198	wk118	wk137	wk050	wk246	tm005	wk390	wk068	wk165	tm096	wk495	wk496
wk062		0.0326	0.0367	0.0351	0.0367	0.0299	0.0334	0.0357	0.0321	0.0362	0.0326	0.0382	0.0386	0.0380	0.0373	0.0383	0.0347	0.0318	0.0387	0.0381	0.0378	0.0362	0.0343	0.0337
wk475	453241		0.0380	0.0283	0.0309	0.0324	0.0332	0.0371	0.0347	0.0368	0.0297	0.0372	0.0360	0.0370	0.0354	0.0387	0.0280	0.0296	0.0331	0.0352	0.0351	0.0290	0.0347	0.0373
wk022	296749	745200		0.0348	0.0382	0.0328	0.0390	0.0362	0.0345	0.0341	0.0360	0.0360	0.0415	0.0379	0.0363	0.0378	0.0401	0.0380	0.0389	0.0406	0.0369	0.0391	0.0359	0.0376
wk245	425757	52361	721037		0.0307	0.0326	0.0320	0.0361	0.0322	0.0349	0.0323	0.0361	0.0351	0.0358	0.0359	0.0369	0.0311	0.0311	0.0335	0.0365	0.0340	0.0297	0.0345	0.0368
wk546	459670	13361	750476	65654		0.0354	0.0343	0.0405	0.0369	0.0405	0.0328	0.0400	0.0399	0.0405	0.0407	0.0412	0.0329	0.0325	0.0393	0.0393	0.0382	0.0320	0.0351	0.0365
tm006	124819	360619	414363	324709	369335		0.0355	0.0325	0.0317	0.0312	0.0325	0.0342	0.0356	0.0355	0.0324	0.0340	0.0355	0.0321	0.0329	0.0360	0.0321	0.0321	0.0297	0.0326
wk391	477191	51188	772303	51469	60165	375618		0.0391	0.0346	0.0390	0.0329	0.0363	0.0391	0.0398	0.0396	0.0378	0.0325	0.0335	0.0396	0.0371	0.0377	0.0363	0.0366	0.0369
wk080	243324	693771	123841	662604	700902	340593	713968		0.0331	0.0333	0.0366	0.0359	0.0387	0.0336	0.0345	0.0352	0.0386	0.0363	0.0354	0.0374	0.0337	0.0356	0.0362	0.0404
wk073	179979	633046	133729	604246	639605	287491	655713	69426		0.0361	0.0348	0.0357	0.0385	0.0348	0.0364	0.0368	0.0346	0.0338	0.0357	0.0367	0.0335	0.0357	0.0346	0.0358
wk009	402572	854863	154034	824356	861736	502424	875747	161912	222885		0.0363	0.0365	0.0382	0.0331	0.0342	0.0364	0.0383	0.0365	0.0336	0.0368	0.0351	0.0346	0.0348	0.0392
wk503	514024	60816	805570	101134	55356	420658	62257	754560	693851	915676		0.0367	0.0376	0.0371	0.0378	0.0354	0.0326	0.0318	0.0368	0.0380	0.0362	0.0324	0.0345	0.0354
wk126	480781	911919	308123	874084	920726	551391	924072	252060	321041	168618	971703		0.0409	0.0339	0.0358	0.0349	0.0355	0.0393	0.0372	0.0350	0.0346	0.0383	0.0362	0.0405
tm198	250408	203225	542262	181118	209301	171640	231515	492337	430376	652730	263888	718172		0.0362	0.0392	0.0377	0.0374	0.0335	0.0354	0.0384	0.0369	0.0333	0.0391	0.0426
wk118	506837	912525	386550	870853	922380	556744	919154	305629	369752	261694	970775	100334	727835		0.0355	0.0330	0.0380	0.0365	0.0347	0.0373	0.0335	0.0360	0.0370	0.0416
wk137	383866	826865	201462	792228	834842	467519	843089	145730	215146	88165	887340	110088	628484	186180		0.0378	0.0381	0.0362	0.0361	0.0370	0.0314	0.0352	0.0350	0.0406
wk050	87191	519317	260397	486387	527049	163401	537627	177193	128010	339036	579946	399485	320516	420657	308046		0.0374	0.0364	0.0367	0.0355	0.0349	0.0362	0.0377	0.0424
wk246	517413	64447	808578	106057	58112	424742	67664	758186	697287	919222	5407	975885	267160	975289	891249	583754		0.0328	0.0359	0.0348	0.0363	0.0310	0.0359	0.0398
tm005	452733	51484	738837	98089	40749	371359	100910	695623	632465	855264	80932	921434	203948	927518	832432	524454	81191		0.0368	0.0378	0.0358	0.0297	0.0363	0.0372
wk390	514238	236015	764332	281121	224205	472655	282917	750656	682229	900282	240733	994805	306727	1017989	895176	598217	237446	184755		0.0341	0.0329	0.0324	0.0351	0.0396
wk068	36112	487882	266188	459246	494552	149133	510713	207466	145215	367274	548693	445042	285458	472883	347760	57472	552161	488283	549806		0.0365	0.0350	0.0366	0.0419
wk165	222228	657368	196824	622298	665544	297597	673134	74026	97370	215947	717736	259258	460403	288361	169962	140404	721699	664210	736443	186946		0.0337	0.0336	0.0400
tm096	542569	100438	829274	149406	89388	457159	116554	785132	722436	945140	54765	1008356	292869	1011372	920926	612884	49443	90441	203599	577974	752142		0.0346	0.0398
wk495	124809	381488	405421	343933	390580	25369	394432	325767	275959	487135	441130	530581	195864	533619	449044	149269	445333	393961	497864	143740	279141	478892		0.0190
wk496	124809	381488	405421	343933	390580	25369	394432	325767	275959	487135	441130	530581	195864	533619	449044	149269	445333	393961	497864	143740	279141	478892	0	

Table S3. Geographic distance (below) and Mash distance (above) for Group 2. Geographic distance is the distance between GPS coordinates (m). Isolation by distance (IBD) was tested by the Mantel test of GenAlex 6.502.

	tm161	tm140	tm020	wk020	wk505	wk292	tm082	tm086	wk028	tm032	tm077	wk006	tm153	tm173	wk295	wk182	tm019	tm022
tm161		0.0348	0.0327	0.0307	0.0290	0.0352	0.0389	0.0305	0.0311	0.0317	0.0391	0.0327	0.0308	0.0413	0.0354	0.0384	0.0357	0.0364
tm140	248966		0.0345	0.0348	0.0344	0.0300	0.0453	0.0344	0.0349	0.0341	0.0313	0.0374	0.0377	0.0418	0.0308	0.0395	0.0373	0.0370
tm020	179375	428327		0.0315	0.0321	0.0348	0.0452	0.0308	0.0311	0.0313	0.0357	0.0371	0.0339	0.0402	0.0365	0.0376	0.0342	0.0311
wk020	59530	274070	170708		0.0284	0.0327	0.0398	0.0298	0.0290	0.0282	0.0351	0.0320	0.0292	0.0413	0.0359	0.0395	0.0377	0.0340
wk505	61918	303635	131131	41544		0.0335	0.0377	0.0286	0.0284	0.0262	0.0361	0.0320	0.0298	0.0407	0.0340	0.0366	0.0350	0.0342
wk292	248753	1779	428124	273498	303236		0.0454	0.0352	0.0335	0.0334	0.0255	0.0374	0.0355	0.0425	0.0310	0.0396	0.0389	0.0361
tm082	76936	298577	152192	24645	33578	297989		0.0394	0.0411	0.0433	0.0482	0.0366	0.0412	0.0523	0.0424	0.0458	0.0407	0.0457
tm086	99711	348664	79802	97137	55624	348440	84341		0.0296	0.0289	0.0369	0.0341	0.0330	0.0394	0.0344	0.0367	0.0334	0.0339
wk028	75845	324256	105084	72291	30944	323986	62631	26025		0.0276	0.0349	0.0339	0.0313	0.0414	0.0347	0.0394	0.0359	0.0336
tm032	61959	303703	131049	41628	84	303305	33641	55539	30863		0.0359	0.0333	0.0317	0.0393	0.0352	0.0389	0.0355	0.0337
tm077	252842	14495	432136	274969	305951	12849	299314	352355	327592	306022		0.0406	0.0368	0.0454	0.0317	0.0436	0.0431	0.0388
wk006	57871	289648	149701	21691	19878	289167	20477	75446	50747	19962	291298		0.0359	0.0410	0.0373	0.0395	0.0379	0.0377
tm153	26180	271804	157189	69775	54697	271678	79971	78674	57753	54703	276325	59495		0.0448	0.0370	0.0411	0.0394	0.0359
tm173	344454	593116	165518	333864	295796	592967	313186	245233	270599	295716	597290	313594	321346		0.0455	0.0297	0.0369	0.0358
wk295	245522	5432	424897	269828	299759	4052	294303	345187	320670	299828	12102	285594	268569	589812		0.0418	0.0388	0.0388
wk182	338302	587035	159242	327339	289365	586877	306601	238996	264305	289285	591144	307109	315300	6714	583711		0.0335	0.0331
tm019	242380	491333	63006	230973	192686	491130	210778	142762	167811	192606	495116	210554	220068	103110	487901	96694		0.0326
tm022	236671	485443	57894	227957	188868	485276	208517	137449	162923	188787	489514	207192	213783	107808	482099	101631	11620	

Table S4. Geographic distance (below) and Mash distance (above) for Group 3. Geographic distance is the distance between GPS coordinates (m). Isolation by distance (IBD) was tested by the Mantel test of GenAlex 6.502.

	tm098	tm150	wk311	tm078	wk172	wk180	wk252	wk215	wk238	tm041	wk211	wk309	tm145	wk242	wk212	wk030	wk171	wk301
tm098		0.0424	0.0372	0.0424	0.0454	0.0427	0.0398	0.0376	0.0472	0.0370	0.0343	0.0364	0.0418	0.0428	0.0350	0.0371	0.0379	0.044597
tm150	712311		0.0398	0.0278	0.0387	0.0342	0.0325	0.0398	0.0388	0.0314	0.0404	0.0403	0.0353	0.0455	0.0403	0.0393	0.0408	0.03188
wk311	350056	364326		0.0405	0.0425	0.0399	0.0326	0.0312	0.0442	0.0319	0.0300	0.0261	0.0428	0.0298	0.0306	0.0259	0.0278	0.0428
tm078	490121	225729	152406		0.0370	0.0348	0.0316	0.0414	0.0400	0.0320	0.0395	0.0409	0.0352	0.0453	0.0398	0.0408	0.0418	0.0254
wk172	823240	119551	478773	333169		0.0282	0.0346	0.0416	0.0323	0.0391	0.0405	0.0417	0.0329	0.0420	0.0388	0.0436	0.0427	0.0342
wk180	826656	125924	483062	336536	9328		0.0343	0.0389	0.0235	0.0353	0.0379	0.0400	0.0238	0.0411	0.0387	0.0404	0.0407	0.0362
wk252	482242	231065	140277	16932	341271	345014		0.0344	0.0374	0.0264	0.0328	0.0340	0.0377	0.0331	0.0325	0.0356	0.0329	0.0315
wk215	310618	428517	88545	233753	546508	551682	219469		0.0423	0.0328	0.0259	0.0339	0.0441	0.0311	0.0290	0.0337	0.0331	0.0437
wk238	848354	147718	505108	358259	28387	22149	366898	573830		0.0404	0.0421	0.0444	0.0258	0.0439	0.0410	0.0439	0.0453	0.0388
tm041	479381	233636	136786	20627	344283	348086	3898	215675	369992		0.0318	0.0336	0.0379	0.0360	0.0348	0.0336	0.0321	0.0360
wk211	304334	438332	98577	244255	556459	561679	229962	10503	583825	226165		0.0307	0.0415	0.0328	0.0271	0.0313	0.0307	0.0420
wk309	353457	361869	6350	152069	476798	481192	139440	86063	503262	135874	96273		0.0423	0.0321	0.0300	0.0272	0.0278	0.0428
tm145	854074	153291	510862	363985	33819	27885	372642	579555	5754	375738	589548	509016		0.0491	0.0415	0.0428	0.0436	0.0365
wk242	780135	99897	439805	290652	53804	52264	300304	511863	71035	303566	522044	438329	76409		0.0330	0.0329	0.0312	0.0448
wk212	308504	431713	91756	237149	549746	554934	222865	3397	577082	219071	7107	89340	582806	515171		0.0316	0.0320	0.0399
wk030	352133	362334	2114	150756	476846	481150	138526	89371	503200	135021	99465	5156	508954	437954	92605		0.0291	0.0459
wk171	369681	342960	26236	127283	456125	460182	115951	114189	482163	112610	124363	28151	487915	416149	117455	25044		0.0438
wk301	490152	225517	152150	864	333120	336510	16195	233348	358243	19921	243851	151779	363970	290694	236745	150493	127073	

Table S5. Marsh distance (below) and SNP-based genetic distance (above) for all individuals. Mash distances were estimated using Mashtree and genetic distances were estimated from Tassel 5 IBD distances. Marsh distance and SNP-based genetic distance were estimates for similarity by Mantel test; GenAlex 6.502). Mash distances were then used to run PCoA in GenAlex 6.502 for comparisons between wild individuals and between wild individuals and cultivars.

	tm153	wk062	tm173	wk182	tm022	wk460	wk461	wk462	wk463	wk464	wk465	wk466	wk467	wk468	wk469	wk470	wk471	wk472	wk473	wk474	wk497	wk498	wk499	wk500	wk501	wk502	wk006	wk475
tm153		0.1379	0.1063	0.1353	0.0833	0.1296	0.1818	0.1628	0.1517	0.1282	0.1860	0.1573	0.1722	0.1667	0.1456	0.1477	0.1667	0.1591	0.1313	0.1552	0.1404	0.1585	0.1190	0.1420	0.1329	0.1667	0.1264	0.1591
wk062	0.0545		0.1063	0.1176	0.0833	0.0679	0.0966	0.0698	0.0843	0.0823	0.0872	0.0805	0.0833	0.0759	0.0886	0.0682	0.0714	0.0739	0.0732	0.0805	0.1348	0.1250	0.1325	0.0562	0.0938	0.0778	0.1494	0.0682
tm173	0.0448	0.0557		0.0741	0.0855	0.1216	0.1420	0.1266	0.0976	0.1042	0.1519	0.1000	0.1386	0.1268	0.1319	0.1111	0.0952	0.1235	0.0959	0.1188	0.1463	0.1467	0.1090	0.1049	0.1042	0.1325	0.1500	0.1235
wk182	0.0411	0.0506	0.0297		0.0789	0.1392	0.1512	0.1429	0.1322	0.1364	0.1607	0.1412	0.1420	0.1316	0.1558	0.1279	0.1087	0.1395	0.1139	0.1235	0.1552	0.1538	0.1325	0.1221	0.1364	0.1420	0.1529	0.1395
tm022	0.0359	0.0543	0.0358	0.0332		0.1067	0.1329	0.1090	0.1125	0.1250	0.1234	0.1090	0.1111	0.1087	0.1164	0.0938	0.0714	0.1013	0.0704	0.1026	0.1000	0.1027	0.1000	0.0823	0.1301	0.1049	0.1090	0.0949
wk460	0.0557	0.0361	0.0497	0.0479	0.0508		0.0732	0.0427	0.0783	0.0625	0.0625	0.0864	0.0595	0.0486	0.0610	0.0671	0.0488	0.0723	0.0541	0.0617	0.1325	0.1333	0.1364	0.0488	0.0864	0.0714	0.1506	0.0542
wk461	0.0543	0.0318	0.0577	0.0525	0.0553	0.0324		0.0690	0.0989	0.0823	0.0862	0.1067	0.0714	0.0741	0.0875	0.0843	0.0521	0.0778	0.0679	0.0682	0.1833	0.1667	0.1667	0.0730	0.0813	0.0824	0.1932	0.0674
wk462	0.0547	0.0374	0.0519	0.0476	0.0514	0.0263	0.0323		0.0852	0.0570	0.0632	0.0814	0.0618	0.0519	0.0625	0.0575	0.0426	0.0632	0.0443	0.0581	0.1591	0.1456	0.1585	0.0517	0.0750	0.0618	0.1802	0.0517
wk463	0.0542	0.0370	0.0506	0.0478	0.0521	0.0289	0.0338	0.0286		0.0688	0.0966	0.0778	0.0870	0.0926	0.0864	0.0889	0.0417	0.0778	0.0732	0.0899	0.1593	0.1524	0.1412	0.0556	0.0741	0.0870	0.1798	0.0778
wk464	0.0535	0.0314	0.0534	0.0491	0.0530	0.0301	0.0293	0.0309	0.0287		0.0714	0.0705	0.0679	0.0725	0.0897	0.0759	0.0256	0.0759	0.0753	0.0696	0.1500	0.1549	0.1267	0.0625	0.0688	0.0741	0.1625	0.0823
wk465	0.0539	0.0350	0.0511	0.0491	0.0511	0.0276	0.0319	0.0294	0.0294	0.0286		0.0872	0.0674	0.0844	0.0705	0.0852	0.0625	0.0862	0.0759	0.0805	0.1761	0.1519	0.1768	0.0632	0.0897	0.0674	0.1919	0.0625
wk466	0.0540	0.0340	0.0524	0.0485	0.0524	0.0288	0.0308	0.0295	0.0300	0.0293	0.0285		0.0944	0.1013	0.0949	0.0909	0.0417	0.0909	0.0813	0.0920	0.1629	0.1463	0.1506	0.0625	0.0823	0.0722	0.1839	0.0795
wk467	0.0536	0.0322	0.0529	0.0489	0.0521	0.0302	0.0281	0.0303	0.0303	0.0270	0.0284	0.0270		0.0741	0.0732	0.0824	0.0408	0.0714	0.0663	0.0611	0.1630	0.1506	0.1570	0.0549	0.0732	0.0645	0.1722	0.0604
wk468	0.0538	0.0350	0.0515	0.0481	0.0509	0.0272	0.0317	0.0292	0.0294	0.0287	0.0290	0.0271	0.0262		0.0714	0.0570	0.0698	0.0688	0.0493	0.0513	0.1625	0.1408	0.1554	0.0570	0.0857	0.0802	0.1731	0.0633
wk469	0.0549	0.0320	0.0538	0.0493	0.0535	0.0295	0.0286	0.0321	0.0302	0.0271	0.0283	0.0290	0.0272	0.0293		0.0813	0.0385	0.0679	0.0764	0.0759	0.1420	0.1370	0.1467	0.0563	0.0949	0.0854	0.1605	0.0679
wk470	0.0545	0.0349	0.0543	0.0494	0.0521	0.0303	0.0299	0.0298	0.0292	0.0295	0.0283	0.0278	0.0275	0.0279	0.0287		0.0745	0.0618	0.0556	0.0562	0.1333	0.1235	0.1412	0.0562	0.0813	0.0769	0.1591	0.0611
wk471	0.0538	0.0346	0.0530	0.0477	0.0524	0.0294	0.0314	0.0295	0.0297	0.0285	0.0288	0.0292	0.0293	0.0281	0.0288	0.0281		0.0612	0.0714	0.0833	0.1250	0.1304	0.1190	0.0408	0.0488	0.0510	0.2065	0.0417
wk472	0.0538	0.0322	0.0521	0.0472	0.0519	0.0286	0.0304	0.0299	0.0276	0.0281	0.0280	0.0297	0.0280	0.0290	0.0278	0.0292	0.0287		0.0617	0.0568	0.1556	0.1463	0.1548	0.0449	0.0625	0.0769	0.1761	0.0556
wk473	0.0541	0.0333	0.0512	0.0482	0.0522	0.0288	0.0317	0.0308	0.0295	0.0299	0.0288	0.0291	0.0287	0.0290	0.0297	0.0295	0.0300	0.0270		0.0549	0.1280	0.1164	0.1118	0.0542	0.0867	0.0663	0.1438	0.0556
wk474	0.0528	0.0340	0.0515	0.0464	0.0511	0.0298	0.0317	0.0294	0.0293	0.0296	0.0300	0.0305	0.0289	0.0301	0.0302	0.0305	0.0300	0.0241	0.0278		0.1461	0.1375	0.1386	0.0618	0.0802	0.0667	0.1552	0.0674
wk497	0.0504	0.0593	0.0523	0.0499	0.0502	0.0585	0.0623	0.0593	0.0570	0.0587	0.0585	0.0574	0.0589	0.0581	0.0585	0.0598	0.0591	0.0570	0.0583	0.0570		0.0723	0.1588	0.1333	0.1605	0.1576	0.1517	0.1556
wk498	0.0507	0.0609	0.0528	0.0515	0.0506	0.0598	0.0637	0.0612	0.0588	0.0609	0.0604	0.0598	0.0616	0.0599	0.0603	0.0617	0.0601	0.0586	0.0603	0.0591	0.0222		0.1579	0.1235	0.1667	0.1386	0.1667	0.1341
wk499	0.0347	0.0565	0.0373	0.0366	0.0341	0.0503	0.0565	0.0478	0.0482	0.0518	0.0505	0.0516	0.0528	0.0505	0.0530	0.0520	0.0510	0.0514	0.0513	0.0496	0.0491	0.0490		0.1369	0.1267	0.1570	0.0783	0.1548
wk500	0.0536	0.0356	0.0489	0.0479	0.0509	0.0262	0.0333	0.0287	0.0273	0.0300	0.0283	0.0277	0.0288	0.0282	0.0294	0.0305	0.0306	0.0275	0.0276	0.0286	0.0583	0.0599	0.0489		0.0732	0.0549	0.1534	0.0449
wk501	0.0540	0.0327	0.0544	0.0516	0.0537	0.0317	0.0297	0.0333	0.0311	0.0283	0.0310	0.0308	0.0288	0.0311	0.0286	0.0317	0.0320	0.0277	0.0291	0.0298	0.0610	0.0614	0.0540	0.0284		0.0732	0.1728	0.0750
wk502	0.0545	0.0329	0.0561	0.0525	0.0556	0.0327	0.0294	0.0361	0.0315	0.0297	0.0323	0.0316	0.0286	0.0328	0.0282	0.0324	0.0326	0.0286	0.0302	0.0297	0.0606	0.0619	0.0557	0.0300	0.0266		0.1722	0.0604
wk006	0.0360	0.0551	0.0410	0.0395	0.0377	0.0519	0.0560	0.0524	0.0508	0.0518	0.0527	0.0525	0.0531	0.0519	0.0520	0.0528	0.0524	0.0516	0.0518	0.0507	0.0519	0.0518	0.0295	0.0513	0.0531	0.0551		0.1761
wk475	0.0545	0.0324	0.0504	0.0470	0.0516	0.0287	0.0312	0.0291	0.0280	0.0295	0.0296	0.0284	0.0289	0.0293	0.0285	0.0294	0.0310	0.0269	0.0287	0.0284	0.0575	0.0596	0.0512	0.0283	0.0294	0.0299	0.0508	
wk022	0.0535	0.0359	0.0532	0.0487	0.0530	0.0389	0.0377	0.0386	0.0373	0.0345	0.0378	0.0359	0.0352	0.0368	0.0369	0.0376	0.0377	0.0367	0.0380	0.0359	0.0580	0.0600	0.0519	0.0372	0.0376	0.0384	0.0531	0.0373
wk245	0.0525	0.0350	0.0522	0.0469	0.0522	0.0308	0.0334	0.0304	0.0274	0.0306	0.0308	0.0308	0.0296	0.0303	0.0304	0.0301	0.0293	0.0284	0.0309	0.0296	0.0565	0.0589	0.0494	0.0303	0.0327	0.0319	0.0506	0.0286
wk546	0.0551	0.0360	0.0558	0.0506	0.0542	0.0316	0.0332	0.0333	0.0300	0.0322	0.0344	0.0322	0.0312	0.0311	0.0314	0.0304	0.0314	0.0303	0.0330	0.0325	0.0589	0.0609	0.0524	0.0311	0.0315	0.0302	0.0530	0.0304
tm006	0.0520	0.0292	0.0515	0.0475	0.0503	0.0318	0.0312	0.0342	0.0327	0.0302	0.0313	0.0313	0.0302	0.0302	0.0309	0.0318	0.0327	0.0303	0.0309	0.0309	0.0578	0.0595	0.0517	0.0310	0.0325	0.0328	0.0523	0.0322
wk391	0.0567	0.0335	0.0559	0.0517	0.0562	0.0350	0.0343	0.0369	0.0335	0.0332	0.0353	0.0351	0.0322	0.0354	0.0333	0.0343	0.0336	0.0315	0.0329	0.0323	0.0609	0.0615	0.0540	0.0349	0.0337	0.0337	0.0507	0.0329
wk238	0.0553	0.0657	0.0609	0.0548	0.0561	0.0663	0.0666	0.0669	0.0633	0.0650	0.0660	0.0660	0.0657	0.0652	0.0650	0.0663	0.0655	0.0642	0.0663	0.0645	0.0451	0.0443	0.0562	0.0675	0.0672	0.0672	0.0550	0.0645
wk080	0.0536	0.0352	0.0504	0.0480	0.0501	0.0351	0.0384	0.0368	0.0360	0.0367	0.0354	0.0356	0.0358	0.0350	0.0359	0.0376	0.0353	0.0349	0.0350	0.0357	0.0578	0.0590	0.0503	0.0339	0.0368	0.0378	0.0522	0.0365
wk073	0.0537	0.0313	0.0532	0.0485	0.0541	0.0352	0.0343	0.0359	0.0346	0.0322	0.0338	0.0340	0.0326	0.0343	0.0327	0.0345	0.0339	0.0331	0.0342	0.0329	0.0576	0.0589	0.0526	0.0341	0.0347	0.0334	0.0526	0.0336
wk009	0.0541	0.0353	0.0512	0.0481	0.0507	0.0351	0.0370	0.0359	0.0351	0.0347	0.0348	0.0344	0.0342	0.0343	0.0349	0.0359	0.0367	0.0348	0.0353	0.0351	0.0587	0.0606	0.0520	0.0330	0.0361	0.0373	0.0518	0.0361
wk503	0.0548	0.0327	0.0547	0.0503	0.0538	0.0320	0.0318	0.0328	0.0318	0.0299	0.0309	0.0296	0.0299	0.0311	0.0291	0.0302	0.0312	0.0295	0.0308	0.0313	0.0588	0.0603	0.0540	0.0307	0.0318	0.0307	0.0529	0.0298
wk171	0.0501	0.0593	0.0518	0.0501	0.0491	0.0594	0.0616	0.0599	0.0565	0.0588	0.0593	0.0583	0.0595	0.0580	0.0590	0.0589	0.0595	0.0563	0.0579	0.0584	0.0291	0.0294	0.0493	0.0592	0.0603	0.0609	0.0519	0.0576
tm082	0.0412	0.0586	0.0523	0.0458	0.0457	0.0596	0.0587	0.0564	0.0556	0.0568	0.0591	0.0572	0.0567	0.0564	0.0560	0.0570	0.0543	0.0551	0.0578	0.0550	0.0545	0.0546	0.0396	0.0586	0.0594	0.0583	0.0366	0.0563
wk020	0.0293	0.0531	0.0413	0.0395	0.0340	0.0532	0.0538	0.0526	0.0524	0.0521	0.0519	0.0522	0.0513	0.0517	0.0516	0.0531	0.0530	0.0513	0.0526	0.0521	0.0493	0.0498	0.0301	0.0509	0.0527	0.0532	0.0320	0.0528
tm086	0.0330	0.0528	0.0395	0.0367	0.0339	0.0480	0.0524	0.0475	0.0473	0.0495	0.0480	0.0478	0.0487	0.0477	0.0506	0.0483	0.0478	0.0480	0.0484	0.0480	0.0478	0.0482	0.0280	0.0471	0.0523	0.0528	0.0341	0.0489
wk126	0.0552	0.0379	0.0502	0.0473	0.0534	0.0371	0.0396	0.0375	0.0369	0.0361	0.0381	0.0362	0.0361	0.0363	0.0364	0.0386	0.0364	0.0351	0.0360	0.0344	0.0587	0.0601	0.0513	0.0363	0.0386	0.0389	0.0504	0.0366
tm198	0.0580	0.0382	0.0543	0.0520	0.0552	0.0352	0.0375	0.0350	0.0350	0.0360	0.0337	0.0344	0.0349	0.0353	0.0352	0.0358	0.0356	0.0342	0.0336	0.0356	0.0612	0.0630	0.0541	0.0329	0.0367	0.0382	0.0567	0.0357
wk118	0.0547	0.0379	0.0501	0.0479	0.0519	0.0361	0.0401	0.0365	0.0353	0.0361	0.0373	0.0360	0.0368	0.0378	0.0370	0.0385	0.0363	0.0349	0.0361	0.0357	0.0603	0.0606	0.0509	0.0345	0.0376	0.0395	0.0532	0.0368
wk137	0.0553	0.0371	0.0511	0.0462	0.0508	0.0336	0.0399	0.0346	0.0346	0.0354	0.0338	0.0334	0.0351	0.0336	0.0377	0.0357	0.0356	0.0337	0.0335	0.0338	0.0574	0.0596	0.0498	0.0337	0.0390	0.0407	0.0524	0.0352
tm150	0.0512	0.0642	0.0565	0.0522	0.0505	0.0629	0.0649	0.0631	0.0605	0.0631	0.0633	0.0626	0.0637	0.0612	0.0629	0.0630	0.0632	0.0623	0.0620	0.0628	0.0434	0.0425	0.0523	0.0637	0.0651	0.0656	0.0531	0.0629
wk050	0.0547	0.0373	0.0523	0.0491	0.0537	0.0373	0.0394	0.0382	0.0376	0.0368	0.0374	0.0368	0.0376	0.0389	0.0369	0.0403	0.0380	0.0356	0.0362	0.0355	0.0611	0.0613	0.0522	0.0360	0.0382	0.0385	0.0529	0.0387
wk246	0.0566	0.0349	0.0501	0.0495	0.0557	0.0301	0.0337	0.0326	0.0302	0.0308	0.0333	0.0332	0.0314	0.0333	0.0301	0.0343	0.0330	0.0282	0.0301	0.0294	0.0593	0.0603	0.0526	0.0297	0.0313	0.0309	0.0518	0.0281
tm041	0.0480	0.0617	0.0515	0.0477	0.0464	0.0572	0.0625	0.0576	0.0564	0.0587	0.0586	0.0569	0.0591	0.0574	0.0596	0.0580	0.0589	0.0584	0.0580	0.0583	0.0350	0.0354	0.0470	0.0585	0.0601	0.0626	0.0496	0.0591
tm077	0.0368	0.0596	0.0454	0.0436	0.0388	0.0576	0.0592	0.0568	0.0552	0.0564	0.0553	0.0570	0.0570	0.0582	0.0586	0.0580	0.0575	0.0563	0.0568	0.0564	0.0431	0.0434	0.0355	0.0555	0.0575	0.0590	0.0406	0.0582
tm078	0.0525	0.0646	0.0566	0.0526	0.0524	0.0637	0.0651	0.0639	0.0609	0.0625	0.0637	0.0635	0.0632	0.0633	0.0631	0.0649	0.0634	0.0626	0.0636	0.0628	0.0438	0.0430	0.0529	0.0637	0.0644	0.0654	0.0546	0.0633
tm140	0.0377	0.0550	0.0418	0.0395	0.0370	0.0523	0.0554	0.0530	0.0512	0.0520	0.0530	0.0528	0.0549	0.0523	0.0542	0.0532	0.0533	0.0529	0.0523	0.0525	0.0409	0.0406	0.0348	0.0512	0.0548	0.0563	0.0374	0.0523
wk252	0.0479	0.0605	0.0508	0.0483	0.0487	0.0585	0.0613	0.0589	0.0562	0.0591	0.0591	0.0583	0.0590	0.0586	0.0580	0.0601	0.0587	0.0568	0.0579	0.0575	0.0340	0.0331	0.0468	0.0580	0.0597	0.0610	0.0484	0.0577
wk292	0.0355	0.0578	0.0426	0.0396	0.0361	0.0553	0.0575	0.0537	0.0537	0.0545	0.0547	0.0558	0.0550	0.0549	0.0557	0.0557	0.0544	0.0543	0.0552	0.0536	0.0424	0.0438	0.0335	0.0536	0.0560	0.0573	0.0374	0.0559
wk295	0.0370	0.0552	0.0455	0.0418	0.0388	0.0548	0.0560	0.0535	0.0517	0.0523	0.0535	0.0534	0.0543	0.0535	0.0542	0.0533	0.0531	0.0532	0.0535	0.0536	0.0414	0.0413	0.0363	0.0537	0.0555	0.0569	0.0373	0.0538
wk301	0.0539	0.0641	0.0574	0.0554	0.0560	0.0657	0.0655	0.0673	0.0626	0.0634	0.0647	0.0651	0.0640	0.0657	0.0628	0.0667	0.0651	0.0626	0.0644	0.0637	0.0421	0.0422	0.0548	0.0657	0.0641	0.0641	0.0534	0.0633
wk172	0.0528	0.0647	0.0561	0.0544	0.0544	0.0651	0.0655	0.0650	0.0613	0.0638	0.0655	0.0644	0.0646	0.0647	0.0625	0.0667	0.0635	0.0628	0.0639	0.0630	0.0416	0.0406	0.0525	0.0649	0.0644	0.0636	0.0513	0.0631
tm005	0.0554	0.0313	0.0543	0.0501	0.0537	0.0295	0.0279	0.0308	0.0309	0.0279	0.0284	0.0281	0.0271	0.0302	0.0283	0.0302	0.0307	0.0283	0.0293	0.0306	0.0601	0.0619	0.0530	0.0282	0.0284	0.0294	0.0535	0.0292
wk180	0.0508	0.0640	0.0539	0.0508	0.0509	0.0621	0.0655	0.0621	0.0599	0.0626	0.0623	0.0614	0.0634	0.0613	0.0623	0.0630	0.0622	0.0611	0.0627	0.0617	0.0414	0.0408	0.0507	0.0630	0.0641	0.0655	0.0507	0.0616
wk242	0.0533	0.0642	0.0516	0.0518	0.0515	0.0603	0.0669	0.0601	0.0579	0.0626	0.0618	0.0611	0.0631	0.0607	0.0616	0.0613	0.0600	0.0596	0.0605	0.0592	0.0320	0.0296	0.0479	0.0604	0.0642	0.0650	0.0516	0.0603
wk390	0.0554	0.0385	0.0507	0.0479	0.0517	0.0328	0.0390	0.0332	0.0326	0.0354	0.0338	0.0344	0.0362	0.0330	0.0362	0.0354	0.0349	0.0333	0.0353	0.0331	0.0582	0.0593	0.0495	0.0307	0.0374	0.0388	0.0533	0.0328
wk068	0.0571	0.0377	0.0504	0.0491	0.0545	0.0347	0.0411	0.0364	0.0358	0.0377	0.0367	0.0374	0.0379	0.0378	0.0368	0.0374	0.0363	0.0339	0.0365	0.0343	0.0600	0.0608	0.0509	0.0335	0.0376	0.0397	0.0507	0.0353
tm032	0.0317	0.0541	0.0393	0.0389	0.0337	0.0500	0.0548	0.0505	0.0507	0.0521	0.0498	0.0514	0.0517	0.0492	0.0533	0.0515	0.0517	0.0512	0.0507	0.0504	0.0488	0.0501	0.0286	0.0488	0.0534	0.0548	0.0333	0.0515
wk505	0.0298	0.0515	0.0407	0.0366	0.0342	0.0519	0.0517	0.0511	0.0507	0.0497	0.0512	0.0510	0.0508	0.0501	0.0505	0.0510	0.0507	0.0495	0.0508	0.0492	0.0475	0.0489	0.0300	0.0504	0.0518	0.0518	0.0320	0.0501
wk165	0.0544	0.0375	0.0492	0.0462	0.0496	0.0337	0.0390	0.0339	0.0353	0.0353	0.0350	0.0332	0.0349	0.0321	0.0358	0.0357	0.0346	0.0331	0.0333	0.0337	0.0573	0.0590	0.0484	0.0319	0.0372	0.0383	0.0501	0.0353
wk028	0.0313	0.0534	0.0414	0.0394	0.0337	0.0514	0.0536	0.0526	0.0507	0.0522	0.0504	0.0520	0.0522	0.0503	0.0528	0.0521	0.0530	0.0511	0.0512	0.0513	0.0498	0.0491	0.0309	0.0495	0.0528	0.0546	0.0339	0.0513
tm098	0.0534	0.0663	0.0591	0.0565	0.0541	0.0651	0.0667	0.0643	0.0633	0.0645	0.0642	0.0627	0.0638	0.0644	0.0645	0.0637	0.0648	0.0632	0.0643	0.0644	0.0381	0.0380	0.0541	0.0645	0.0659	0.0671	0.0569	0.0655
wk030	0.0514	0.0609	0.0552	0.0532	0.0494	0.0594	0.0613	0.0606	0.0583	0.0601	0.0593	0.0592	0.0597	0.0594	0.0594	0.0600	0.0607	0.0581	0.0581	0.0596	0.0304	0.0308	0.0520	0.0602	0.0608	0.0629	0.0531	0.0586
wk309	0.0501	0.0601	0.0541	0.0518	0.0500	0.0618	0.0604	0.0623	0.0588	0.0593	0.0600	0.0588	0.0599	0.0602	0.0600	0.0603	0.0602	0.0577	0.0594	0.0593	0.0295	0.0298	0.0518	0.0610	0.0611	0.0604	0.0529	0.0599
wk311	0.0510	0.0605	0.0543	0.0518	0.0501	0.0600	0.0612	0.0595	0.0576	0.0587	0.0579	0.0571	0.0595	0.0584	0.0585	0.0577	0.0588	0.0571	0.0589	0.0581	0.0277	0.0280	0.0503	0.0590	0.0602	0.0615	0.0525	0.0588
tm020	0.0339	0.0553	0.0402	0.0376	0.0311	0.0519	0.0549	0.0518	0.0526	0.0533	0.0514	0.0521	0.0533	0.0523	0.0543	0.0533	0.0529	0.0527	0.0514	0.0511	0.0483	0.0495	0.0332	0.0511	0.0551	0.0564	0.0371	0.0530
tm019	0.0394	0.0541	0.0369	0.0335	0.0326	0.0486	0.0545	0.0483	0.0482	0.0512	0.0497	0.0495	0.0500	0.0472	0.0508	0.0491	0.0492	0.0487	0.0494	0.0483	0.0491	0.0505	0.0336	0.0485	0.0533	0.0547	0.0379	0.0486
tm161	0.0308	0.0521	0.0413	0.0384	0.0364	0.0512	0.0527	0.0515	0.0498	0.0504	0.0526	0.0515	0.0512	0.0498	0.0522	0.0515	0.0521	0.0508	0.0522	0.0505	0.0460	0.0461	0.0329	0.0519	0.0530	0.0528	0.0327	0.0503
tm096	0.0542	0.0352	0.0505	0.0467	0.0506	0.0282	0.0338	0.0290	0.0283	0.0306	0.0298	0.0299	0.0291	0.0302	0.0308	0.0295	0.0297	0.0282	0.0298	0.0299	0.0587	0.0606	0.0500	0.0259	0.0293	0.0320	0.0516	0.0291
tm145	0.0529	0.0663	0.0594	0.0543	0.0537	0.0669	0.0662	0.0685	0.0646	0.0653	0.0663	0.0655	0.0655	0.0669	0.0654	0.0669	0.0667	0.0663	0.0661	0.0665	0.0442	0.0451	0.0556	0.0669	0.0662	0.0673	0.0563	0.0666
wk495	0.0532	0.0336	0.0527	0.0484	0.0526	0.0340	0.0355	0.0349	0.0329	0.0333	0.0342	0.0336	0.0327	0.0329	0.0343	0.0332	0.0338	0.0320	0.0346	0.0324	0.0571	0.0595	0.0516	0.0342	0.0362	0.0353	0.0524	0.0346
wk496	0.0554	0.0336	0.0581	0.0519	0.0572	0.0395	0.0357	0.0400	0.0373	0.0361	0.0386	0.0368	0.0349	0.0377	0.0359	0.0371	0.0371	0.0342	0.0381	0.0356	0.0606	0.0621	0.0569	0.0395	0.0372	0.0354	0.0558	0.0370
wk211	0.0492	0.0603	0.0499	0.0485	0.0472	0.0574	0.0618	0.0568	0.0553	0.0582	0.0574	0.0569	0.0582	0.0568	0.0579	0.0578	0.0577	0.0562	0.0572	0.0567	0.0306	0.0300	0.0454	0.0567	0.0602	0.0606	0.0517	0.0583
wk212	0.0505	0.0591	0.0523	0.0513	0.0505	0.0598	0.0603	0.0606	0.0575	0.0574	0.0600	0.0591	0.0583	0.0591	0.0574	0.0593	0.0586	0.0568	0.0584	0.0578	0.0307	0.0294	0.0499	0.0601	0.0596	0.0586	0.0507	0.0583
wk215	0.0518	0.0619	0.0513	0.0485	0.0495	0.0579	0.0624	0.0559	0.0546	0.0601	0.0579	0.0582	0.0599	0.0574	0.0599	0.0580	0.0569	0.0569	0.0580	0.0570	0.0334	0.0323	0.0464	0.0575	0.0623	0.0627	0.0514	0.0573

	wk022	wk245	wk546	tm006	wk391	wk238	wk080	wk073	wk009	wk503	wk171	tm082	wk020	tm086	wk126	tm198	wk118	wk137	tm150	wk050	wk246	tm041	tm077	tm078	tm140	wk252	wk292
tm153	0.1579	0.1744	0.1456	0.1591	0.1548	0.1131	0.1500	0.1494	0.1647	0.1798	0.1603	0.1284	0.1063	0.1429	0.1494	0.1688	0.1628	0.1494	0.1000	0.1543	0.1573	0.1839	0.1173	0.1216	0.1316	0.1591	0.1014
wk062	0.0965	0.1047	0.0633	0.0852	0.0783	0.1084	0.0778	0.0833	0.0765	0.0944	0.1474	0.1667	0.1013	0.0795	0.0747	0.0875	0.0920	0.0805	0.1053	0.0556	0.0611	0.1667	0.1358	0.1081	0.0921	0.1307	0.0959
tm173	0.0833	0.1203	0.1081	0.1049	0.0987	0.1218	0.1145	0.0845	0.1266	0.1463	0.1284	0.1719	0.0779	0.0641	0.1125	0.1370	0.1250	0.1125	0.0882	0.1216	0.1220	0.1688	0.0946	0.1233	0.0972	0.1235	0.0714
wk182	0.1204	0.1548	0.1266	0.1337	0.1235	0.1296	0.1193	0.1067	0.1386	0.1494	0.1623	0.1857	0.1013	0.1071	0.1176	0.1410	0.1310	0.1294	0.1233	0.1266	0.1379	0.1802	0.1250	0.1370	0.1067	0.1379	0.0833
tm022	0.0761	0.1364	0.0786	0.1139	0.1216	0.0616	0.0926	0.1014	0.1053	0.1188	0.1232	0.1129	0.0743	0.0541	0.0897	0.1181	0.0962	0.0949	0.0672	0.0972	0.0938	0.1266	0.0903	0.0725	0.0694	0.0949	0.0735
wk460	0.0900	0.1049	0.0479	0.1024	0.0779	0.1053	0.0714	0.0563	0.0633	0.0723	0.1597	0.1791	0.1096	0.0714	0.0732	0.0676	0.0938	0.0864	0.1087	0.0600	0.0542	0.1790	0.1467	0.1029	0.1071	0.1402	0.0970
wk461	0.0862	0.1034	0.0813	0.0955	0.0824	0.1566	0.0769	0.0705	0.0756	0.0778	0.1899	0.2361	0.1375	0.1136	0.0682	0.0802	0.0862	0.1023	0.1447	0.0671	0.0833	0.2216	0.1607	0.1667	0.1364	0.1742	0.1419
wk462	0.0893	0.0882	0.0449	0.0977	0.0732	0.1296	0.0843	0.0577	0.0581	0.0795	0.1688	0.2143	0.1282	0.1000	0.0756	0.0556	0.0882	0.0640	0.1351	0.0671	0.0625	0.2035	0.1625	0.1438	0.1200	0.1552	0.1319
wk463	0.0776	0.0852	0.0741	0.0778	0.0706	0.1369	0.0924	0.0759	0.0805	0.1099	0.1500	0.2192	0.1173	0.0795	0.0843	0.0976	0.0966	0.0955	0.1104	0.0843	0.0769	0.1966	0.1488	0.1447	0.1218	0.1500	0.1133
wk464	0.0833	0.0909	0.0694	0.0823	0.0608	0.1233	0.0926	0.0580	0.0658	0.0938	0.1522	0.2031	0.1127	0.0833	0.0833	0.0704	0.0909	0.0962	0.0985	0.0833	0.0813	0.1923	0.1319	0.1308	0.1269	0.1519	0.0938
wk465	0.1034	0.1000	0.0696	0.0805	0.1084	0.1481	0.0843	0.0823	0.0747	0.0739	0.1818	0.2357	0.1474	0.1429	0.0756	0.0915	0.0941	0.0930	0.1351	0.0783	0.0739	0.2209	0.1750	0.1644	0.1333	0.1667	0.1486
wk466	0.0862	0.1105	0.0759	0.0625	0.1024	0.1369	0.0944	0.0779	0.0647	0.1011	0.1474	0.2123	0.1266	0.1071	0.0805	0.1063	0.1047	0.0977	0.1267	0.0926	0.0843	0.2069	0.1524	0.1554	0.1250	0.1477	0.1233
wk467	0.0862	0.1011	0.0610	0.0879	0.0814	0.1412	0.0753	0.0750	0.0739	0.0652	0.1728	0.2162	0.1280	0.1136	0.0667	0.0783	0.0674	0.0889	0.1410	0.0655	0.0598	0.2056	0.1488	0.1494	0.1139	0.1538	0.1250
wk468	0.0849	0.1000	0.0571	0.1013	0.0800	0.1284	0.0617	0.0704	0.0592	0.0679	0.1522	0.2121	0.1286	0.0811	0.0513	0.0634	0.0779	0.0897	0.1343	0.0694	0.0741	0.1859	0.1447	0.1397	0.0970	0.1563	0.1250
wk469	0.0833	0.0949	0.0634	0.1111	0.0933	0.1081	0.0915	0.0725	0.0649	0.0864	0.1643	0.1923	0.1268	0.1061	0.0875	0.0694	0.0962	0.0823	0.1232	0.0667	0.0679	0.1962	0.1644	0.1136	0.1250	0.1563	0.1154
wk470	0.0789	0.1034	0.0432	0.0955	0.0774	0.1024	0.0824	0.0769	0.0756	0.0833	0.1456	0.1875	0.1188	0.0698	0.0682	0.0741	0.0852	0.0795	0.0974	0.0732	0.0667	0.1705	0.1402	0.1267	0.1026	0.1404	0.1133
wk471	0.0854	0.0435	0.0395	0.0510	0.0556	0.1705	0.0612	0.0435	0.0625	0.0510	0.1364	0.2558	0.0978	0.1250	0.0714	0.0652	0.0532	0.0612	0.1477	0.0532	0.0408	0.1489	0.1304	0.1628	0.0889	0.0833	0.1136
wk472	0.0862	0.0852	0.0500	0.0944	0.0765	0.1325	0.0879	0.0833	0.0698	0.0778	0.1709	0.2014	0.1313	0.0930	0.0787	0.0679	0.0747	0.0682	0.1316	0.0610	0.0611	0.1989	0.1627	0.1467	0.1234	0.1517	0.1351
wk473	0.0862	0.1076	0.0486	0.0802	0.0658	0.1000	0.0602	0.0625	0.0513	0.0793	0.1458	0.1940	0.0903	0.0795	0.0563	0.0685	0.0679	0.0625	0.1103	0.0608	0.0671	0.1813	0.1149	0.1045	0.0845	0.1296	0.0758
wk474	0.0948	0.1105	0.0500	0.0909	0.0663	0.1159	0.0667	0.0705	0.0588	0.0674	0.1603	0.1901	0.1139	0.0795	0.0517	0.0625	0.0690	0.0805	0.1067	0.0679	0.0730	0.1897	0.1296	0.1351	0.0987	0.1477	0.1081
wk497	0.1754	0.1875	0.1358	0.1667	0.1628	0.0833	0.1413	0.1582	0.1552	0.1703	0.1188	0.1757	0.1235	0.1364	0.1404	0.1524	0.1477	0.1404	0.0974	0.1506	0.1484	0.1124	0.1386	0.0789	0.0962	0.1056	0.1000
wk498	0.1667	0.1750	0.1319	0.1463	0.1623	0.0886	0.1205	0.1357	0.1346	0.1402	0.0959	0.1985	0.1382	0.1711	0.1220	0.1438	0.1392	0.1313	0.1071	0.1284	0.1341	0.1375	0.1486	0.1000	0.1111	0.1049	0.1159
wk499	0.1373	0.1646	0.1494	0.1488	0.1375	0.1282	0.1337	0.1301	0.1543	0.1647	0.1622	0.1029	0.1090	0.1163	0.1265	0.1582	0.1446	0.1506	0.1111	0.1429	0.1529	0.1928	0.1234	0.1429	0.1250	0.1726	0.0833
wk500	0.0776	0.0747	0.0375	0.0674	0.0655	0.1084	0.0714	0.0641	0.0523	0.0778	0.1456	0.1806	0.1063	0.0909	0.0625	0.0617	0.0682	0.0625	0.1053	0.0488	0.0444	0.1761	0.1402	0.1133	0.0909	0.1292	0.1014
wk501	0.0700	0.0897	0.0634	0.0750	0.0667	0.1351	0.1037	0.0786	0.0844	0.0926	0.1643	0.2154	0.1197	0.0972	0.0886	0.0833	0.0886	0.0886	0.1045	0.0822	0.0802	0.2025	0.1438	0.1515	0.1471	0.1625	0.1154
wk502	0.0776	0.1067	0.0549	0.0659	0.0930	0.1294	0.0806	0.0750	0.0625	0.0761	0.1605	0.2095	0.1341	0.1250	0.0667	0.0783	0.0730	0.0833	0.1282	0.0714	0.0652	0.2000	0.1548	0.1494	0.1203	0.1484	0.1316
wk006	0.2091	0.2035	0.1519	0.1818	0.1747	0.1265	0.1500	0.1753	0.1706	0.1798	0.1962	0.0479	0.1203	0.1098	0.1437	0.1438	0.1628	0.1724	0.1316	0.1667	0.1685	0.1954	0.1358	0.1284	0.1118	0.1761	0.0685
wk475	0.0776	0.0909	0.0494	0.0778	0.0833	0.1265	0.0714	0.0641	0.0698	0.0611	0.1582	0.2083	0.1250	0.1047	0.0674	0.0802	0.0805	0.0739	0.1118	0.0488	0.0444	0.1989	0.1646	0.1400	0.1169	0.1461	0.1267
wk022		0.0727	0.0673	0.0702	0.0755	0.1346	0.0862	0.0556	0.0789	0.0877	0.1509	0.2553	0.1277	0.1452	0.0714	0.0962	0.0804	0.0727	0.1122	0.0755	0.0789	0.2000	0.1404	0.1429	0.1196	0.1316	0.1277
wk245	0.0345		0.0962	0.1080	0.0854	0.1667	0.1180	0.0789	0.0952	0.1193	0.1753	0.2429	0.1474	0.1250	0.1092	0.0886	0.1118	0.1047	0.1351	0.0813	0.0909	0.2267	0.1688	0.1711	0.1467	0.1782	0.1458
wk546	0.0378	0.0300		0.0813	0.0733	0.1149	0.0793	0.0652	0.0584	0.0741	0.1571	0.1746	0.1233	0.0875	0.0633	0.0625	0.0641	0.0570	0.0970	0.0616	0.0494	0.1835	0.1438	0.1212	0.1029	0.1313	0.1061
tm006	0.0326	0.0322	0.0346		0.0952	0.1386	0.0824	0.0769	0.0756	0.0833	0.1519	0.2260	0.1250	0.1047	0.0722	0.1049	0.0920	0.1023	0.1169	0.0783	0.0833	0.2045	0.1463	0.1579	0.1299	0.1517	0.1216
wk391	0.0387	0.0315	0.0343	0.0355		0.1410	0.0930	0.0733	0.0976	0.1059	0.1622	0.2153	0.1118	0.0682	0.0843	0.0844	0.0915	0.1024	0.1111	0.0759	0.0824	0.2048	0.1410	0.1479	0.1181	0.1607	0.1127
wk238	0.0651	0.0629	0.0633	0.0646	0.0633		0.1118	0.1096	0.1235	0.1412	0.1026	0.1449	0.1081	0.1892	0.1098	0.1400	0.1220	0.1220	0.0411	0.1184	0.1235	0.1220	0.1250	0.0556	0.1056	0.1145	0.0942
wk080	0.0359	0.0358	0.0398	0.0321	0.0390	0.0661		0.0688	0.0682	0.0652	0.1481	0.1959	0.1037	0.0909	0.0444	0.0904	0.0730	0.0944	0.1154	0.0655	0.0815	0.1833	0.1190	0.1234	0.0886	0.1374	0.0987
wk073	0.0341	0.0320	0.0361	0.0313	0.0339	0.0629	0.0330		0.0688	0.0759	0.1232	0.2154	0.1014	0.1000	0.0649	0.0811	0.0897	0.0714	0.0909	0.0724	0.0759	0.1859	0.1338	0.1288	0.0985	0.1346	0.1000
wk009	0.0336	0.0345	0.0391	0.0300	0.0393	0.0682	0.0327	0.0350		0.0747	0.1558	0.2101	0.1299	0.1250	0.0529	0.0671	0.0765	0.0647	0.1301	0.0723	0.0805	0.1941	0.1456	0.1438	0.1149	0.1512	0.1301
wk503	0.0358	0.0321	0.0324	0.0322	0.0330	0.0658	0.0367	0.0341	0.0357		0.1750	0.2230	0.1420	0.1364	0.0562	0.0915	0.0795	0.1011	0.1474	0.0723	0.0761	0.2079	0.1566	0.1645	0.1282	0.1611	0.1400
wk171	0.0582	0.0573	0.0585	0.0565	0.0611	0.0453	0.0580	0.0584	0.0589	0.0596		0.2258	0.1429	0.1923	0.1474	0.1690	0.1603	0.1474	0.1216	0.1528	0.1563	0.1474	0.1579	0.1250	0.1397	0.1329	0.1324
tm082	0.0557	0.0524	0.0534	0.0584	0.0541	0.0535	0.0578	0.0557	0.0583	0.0567	0.0558		0.1385	0.1389	0.1918	0.1719	0.2071	0.1972	0.1508	0.1912	0.1959	0.2113	0.1615	0.1475	0.1066	0.1944	0.1034
wk020	0.0511	0.0523	0.0545	0.0504	0.0561	0.0556	0.0506	0.0515	0.0519	0.0527	0.0487	0.0398		0.0814	0.1063	0.1319	0.1154	0.1203	0.0896	0.1096	0.1235	0.1582	0.0890	0.1096	0.0867	0.1250	0.0753
tm086	0.0476	0.0468	0.0489	0.0474	0.0524	0.0546	0.0478	0.0497	0.0471	0.0509	0.0471	0.0394	0.0298		0.0833	0.1220	0.1190	0.1250	0.1282	0.0897	0.1023	0.1463	0.0930	0.1316	0.0714	0.1163	0.0952
wk126	0.0357	0.0354	0.0389	0.0341	0.0363	0.0633	0.0355	0.0348	0.0358	0.0365	0.0586	0.0547	0.0529	0.0490		0.0750	0.0698	0.0862	0.1118	0.0549	0.0730	0.1839	0.1173	0.1200	0.0921	0.1364	0.1027
tm198	0.0410	0.0351	0.0392	0.0350	0.0386	0.0704	0.0383	0.0377	0.0377	0.0371	0.0609	0.0618	0.0550	0.0514	0.0406		0.0823	0.0783	0.1397	0.0769	0.0854	0.1938	0.1554	0.1567	0.1111	0.1667	0.1357
wk118	0.0373	0.0355	0.0401	0.0354	0.0397	0.0687	0.0338	0.0348	0.0329	0.0377	0.0598	0.0579	0.0528	0.0487	0.0338	0.0362		0.0756	0.1267	0.0750	0.0795	0.1860	0.1375	0.1351	0.0987	0.1494	0.1111
wk137	0.0363	0.0356	0.0394	0.0323	0.0394	0.0654	0.0343	0.0351	0.0339	0.0374	0.0576	0.0577	0.0529	0.0467	0.0357	0.0391	0.0353		0.1333	0.0679	0.0787	0.1839	0.1605	0.1351	0.1118	0.1477	0.1301
tm150	0.0635	0.0617	0.0626	0.0605	0.0652	0.0389	0.0627	0.0634	0.0631	0.0645	0.0408	0.0596	0.0520	0.0496	0.0648	0.0670	0.0652	0.0612		0.1284	0.1154	0.1000	0.1301	0.0538	0.1231	0.1118	0.0781
wk050	0.0377	0.0371	0.0406	0.0345	0.0381	0.0674	0.0355	0.0366	0.0363	0.0360	0.0599	0.0569	0.0526	0.0501	0.0350	0.0376	0.0336	0.0377	0.0669		0.0602	0.1852	0.1533	0.1268	0.1071	0.1463	0.1159
wk246	0.0401	0.0314	0.0328	0.0353	0.0325	0.0664	0.0387	0.0338	0.0378	0.0329	0.0614	0.0585	0.0553	0.0520	0.0353	0.0376	0.0381	0.0380	0.0666	0.0375		0.1798	0.1566	0.1250	0.1090	0.1389	0.1200
tm041	0.0587	0.0564	0.0591	0.0569	0.0626	0.0404	0.0582	0.0590	0.0589	0.0606	0.0321	0.0552	0.0488	0.0448	0.0610	0.0625	0.0604	0.0555	0.0314	0.0629	0.0621		0.1829	0.1081	0.1429	0.0955	0.1370
tm077	0.0557	0.0566	0.0587	0.0556	0.0608	0.0545	0.0559	0.0577	0.0553	0.0596	0.0411	0.0482	0.0351	0.0369	0.0584	0.0579	0.0550	0.0565	0.0478	0.0566	0.0603	0.0417		0.1176	0.0915	0.1548	0.0857
tm078	0.0629	0.0619	0.0647	0.0608	0.0658	0.0400	0.0636	0.0632	0.0635	0.0643	0.0418	0.0604	0.0514	0.0512	0.0620	0.0671	0.0648	0.0619	0.0278	0.0669	0.0660	0.0320	0.0497		0.1000	0.0933	0.0956
tm140	0.0531	0.0527	0.0543	0.0513	0.0564	0.0497	0.0524	0.0527	0.0525	0.0543	0.0387	0.0453	0.0348	0.0344	0.0527	0.0559	0.0538	0.0503	0.0451	0.0547	0.0553	0.0386	0.0313	0.0451		0.1026	0.0915
wk252	0.0591	0.0566	0.0590	0.0576	0.0592	0.0374	0.0585	0.0580	0.0598	0.0593	0.0329	0.0510	0.0486	0.0459	0.0575	0.0618	0.0601	0.0575	0.0325	0.0592	0.0591	0.0264	0.0414	0.0316	0.0385		0.1014
wk292	0.0544	0.0541	0.0565	0.0531	0.0583	0.0517	0.0532	0.0550	0.0537	0.0576	0.0411	0.0454	0.0327	0.0352	0.0553	0.0564	0.0532	0.0540	0.0464	0.0544	0.0581	0.0408	0.0255	0.0476	0.0300	0.0416	
wk295	0.0532	0.0519	0.0528	0.0525	0.0553	0.0484	0.0522	0.0532	0.0534	0.0555	0.0398	0.0424	0.0359	0.0344	0.0534	0.0567	0.0542	0.0519	0.0456	0.0553	0.0575	0.0393	0.0317	0.0473	0.0308	0.0391	0.0310
wk301	0.0643	0.0630	0.0637	0.0637	0.0648	0.0388	0.0656	0.0625	0.0664	0.0638	0.0438	0.0574	0.0532	0.0544	0.0635	0.0700	0.0674	0.0649	0.0319	0.0675	0.0636	0.0360	0.0532	0.0254	0.0485	0.0315	0.0524
wk172	0.0637	0.0618	0.0620	0.0642	0.0623	0.0323	0.0655	0.0629	0.0657	0.0628	0.0427	0.0542	0.0527	0.0530	0.0616	0.0695	0.0676	0.0656	0.0388	0.0664	0.0630	0.0391	0.0516	0.0370	0.0472	0.0346	0.0500
tm005	0.0368	0.0307	0.0319	0.0317	0.0332	0.0671	0.0358	0.0331	0.0356	0.0313	0.0600	0.0599	0.0525	0.0515	0.0390	0.0331	0.0367	0.0354	0.0648	0.0366	0.0324	0.0610	0.0586	0.0654	0.0546	0.0602	0.0561
wk180	0.0628	0.0596	0.0616	0.0608	0.0637	0.0235	0.0632	0.0619	0.0626	0.0637	0.0407	0.0546	0.0503	0.0483	0.0620	0.0673	0.0648	0.0605	0.0342	0.0656	0.0637	0.0353	0.0493	0.0348	0.0448	0.0343	0.0468
wk242	0.0618	0.0586	0.0613	0.0614	0.0609	0.0439	0.0608	0.0608	0.0620	0.0613	0.0312	0.0509	0.0519	0.0477	0.0589	0.0648	0.0625	0.0582	0.0455	0.0617	0.0607	0.0360	0.0461	0.0453	0.0418	0.0331	0.0448
wk390	0.0386	0.0333	0.0387	0.0327	0.0395	0.0680	0.0351	0.0349	0.0332	0.0369	0.0597	0.0582	0.0529	0.0474	0.0368	0.0352	0.0345	0.0357	0.0655	0.0369	0.0355	0.0601	0.0575	0.0659	0.0520	0.0602	0.0540
wk068	0.0397	0.0362	0.0389	0.0357	0.0368	0.0654	0.0372	0.0355	0.0368	0.0376	0.0604	0.0581	0.0545	0.0485	0.0351	0.0383	0.0375	0.0367	0.0648	0.0356	0.0347	0.0609	0.0583	0.0659	0.0528	0.0585	0.0550
tm032	0.0526	0.0510	0.0538	0.0502	0.0559	0.0571	0.0497	0.0521	0.0496	0.0538	0.0491	0.0433	0.0282	0.0289	0.0531	0.0548	0.0518	0.0497	0.0507	0.0540	0.0543	0.0462	0.0359	0.0513	0.0341	0.0472	0.0334
wk505	0.0496	0.0487	0.0507	0.0486	0.0524	0.0508	0.0493	0.0498	0.0503	0.0515	0.0471	0.0378	0.0284	0.0286	0.0515	0.0541	0.0513	0.0503	0.0494	0.0526	0.0534	0.0457	0.0361	0.0492	0.0344	0.0447	0.0335
wk165	0.0371	0.0344	0.0380	0.0323	0.0381	0.0658	0.0336	0.0335	0.0346	0.0365	0.0573	0.0553	0.0517	0.0465	0.0350	0.0366	0.0342	0.0311	0.0614	0.0353	0.0369	0.0568	0.0565	0.0618	0.0502	0.0573	0.0533
wk028	0.0512	0.0504	0.0532	0.0494	0.0553	0.0554	0.0507	0.0521	0.0500	0.0540	0.0476	0.0411	0.0290	0.0296	0.0525	0.0549	0.0519	0.0496	0.0494	0.0532	0.0551	0.0446	0.0349	0.0502	0.0349	0.0465	0.0335
tm098	0.0643	0.0628	0.0654	0.0615	0.0678	0.0472	0.0647	0.0645	0.0651	0.0647	0.0379	0.0615	0.0537	0.0525	0.0660	0.0668	0.0661	0.0633	0.0424	0.0663	0.0680	0.0370	0.0473	0.0424	0.0468	0.0398	0.0469
wk030	0.0590	0.0587	0.0609	0.0562	0.0619	0.0439	0.0589	0.0604	0.0586	0.0598	0.0292	0.0600	0.0504	0.0496	0.0603	0.0630	0.0616	0.0572	0.0393	0.0623	0.0624	0.0336	0.0427	0.0408	0.0407	0.0356	0.0427
wk309	0.0581	0.0585	0.0594	0.0572	0.0618	0.0444	0.0590	0.0592	0.0598	0.0593	0.0278	0.0559	0.0493	0.0492	0.0608	0.0631	0.0625	0.0587	0.0403	0.0623	0.0628	0.0336	0.0431	0.0409	0.0406	0.0340	0.0427
wk311	0.0592	0.0571	0.0592	0.0565	0.0614	0.0442	0.0584	0.0586	0.0582	0.0581	0.0278	0.0554	0.0500	0.0484	0.0595	0.0622	0.0617	0.0569	0.0398	0.0609	0.0617	0.0319	0.0415	0.0405	0.0395	0.0326	0.0415
tm020	0.0537	0.0516	0.0541	0.0503	0.0572	0.0552	0.0524	0.0549	0.0518	0.0547	0.0476	0.0452	0.0315	0.0308	0.0541	0.0563	0.0538	0.0509	0.0492	0.0536	0.0552	0.0452	0.0357	0.0509	0.0346	0.0471	0.0348
tm019	0.0494	0.0477	0.0497	0.0487	0.0535	0.0541	0.0485	0.0503	0.0481	0.0514	0.0472	0.0407	0.0377	0.0334	0.0489	0.0532	0.0517	0.0471	0.0507	0.0519	0.0520	0.0478	0.0431	0.0528	0.0373	0.0476	0.0389
tm161	0.0508	0.0487	0.0515	0.0497	0.0535	0.0516	0.0512	0.0509	0.0519	0.0519	0.0459	0.0389	0.0307	0.0305	0.0509	0.0563	0.0522	0.0513	0.0492	0.0535	0.0522	0.0454	0.0391	0.0496	0.0348	0.0452	0.0352
tm096	0.0383	0.0295	0.0316	0.0316	0.0358	0.0665	0.0348	0.0347	0.0336	0.0320	0.0588	0.0571	0.0520	0.0481	0.0377	0.0331	0.0358	0.0345	0.0632	0.0363	0.0311	0.0577	0.0566	0.0632	0.0515	0.0582	0.0547
tm145	0.0650	0.0647	0.0651	0.0641	0.0677	0.0259	0.0666	0.0642	0.0656	0.0660	0.0436	0.0615	0.0524	0.0534	0.0665	0.0697	0.0680	0.0669	0.0353	0.0684	0.0680	0.0379	0.0510	0.0352	0.0486	0.0377	0.0490
wk495	0.0350	0.0344	0.0349	0.0293	0.0363	0.0636	0.0355	0.0339	0.0340	0.0339	0.0567	0.0547	0.0533	0.0466	0.0359	0.0386	0.0371	0.0343	0.0620	0.0377	0.0355	0.0573	0.0568	0.0625	0.0538	0.0586	0.0559
wk496	0.0371	0.0369	0.0359	0.0324	0.0373	0.0650	0.0396	0.0353	0.0383	0.0354	0.0606	0.0562	0.0552	0.0512	0.0394	0.0422	0.0416	0.0403	0.0660	0.0414	0.0394	0.0614	0.0608	0.0650	0.0575	0.0618	0.0600
wk211	0.0575	0.0549	0.0580	0.0573	0.0618	0.0421	0.0563	0.0579	0.0583	0.0583	0.0308	0.0555	0.0479	0.0451	0.0598	0.0593	0.0594	0.0568	0.0404	0.0603	0.0610	0.0318	0.0426	0.0395	0.0390	0.0328	0.0410
wk212	0.0586	0.0576	0.0586	0.0567	0.0589	0.0410	0.0589	0.0576	0.0605	0.0570	0.0320	0.0532	0.0481	0.0488	0.0577	0.0620	0.0604	0.0586	0.0403	0.0595	0.0588	0.0348	0.0451	0.0398	0.0415	0.0325	0.0427
wk215	0.0579	0.0552	0.0576	0.0581	0.0628	0.0423	0.0576	0.0588	0.0587	0.0597	0.0331	0.0540	0.0518	0.0463	0.0593	0.0599	0.0589	0.0550	0.0398	0.0620	0.0603	0.0328	0.0453	0.0414	0.0399	0.0344	0.0437

	wk295	wk301	wk172	tm005	wk180	wk242	wk390	wk068	tm032	wk505	wk165	wk028	tm098	wk030	wk309	wk311	tm020	tm019	tm161	tm096	tm145	wk495	wk496	wk211	wk212	wk215
tm153	0.1090	0.1481	0.0949	0.1667	0.0882	0.1629	0.1379	0.1623	0.0921	0.1167	0.1570	0.1092	0.1441	0.1585	0.1867	0.1563	0.0952	0.0864	0.0563	0.1628	0.1042	0.1556	0.1714	0.1104	0.1500	0.1386
wk062	0.1026	0.1235	0.0886	0.0833	0.1012	0.1180	0.0575	0.0886	0.0909	0.1124	0.0739	0.1322	0.1271	0.0915	0.1543	0.1250	0.1829	0.1173	0.1029	0.0747	0.1181	0.0722	0.0704	0.1039	0.1667	0.1341
tm173	0.0986	0.1233	0.1000	0.1325	0.0789	0.1506	0.1000	0.1357	0.0956	0.0976	0.1203	0.0854	0.1207	0.1299	0.1554	0.1400	0.1282	0.0676	0.0794	0.1266	0.0882	0.1205	0.1349	0.1319	0.1506	0.1533
wk182	0.1169	0.1346	0.1090	0.1420	0.1049	0.1667	0.1118	0.1316	0.1081	0.1207	0.1369	0.1264	0.1724	0.1375	0.1772	0.1667	0.1548	0.1013	0.0833	0.1369	0.1197	0.1364	0.1377	0.1382	0.1818	0.1605
tm022	0.0571	0.0845	0.0423	0.1173	0.0608	0.1188	0.0641	0.0956	0.0588	0.0750	0.0897	0.0897	0.1053	0.0921	0.1389	0.1216	0.0959	0.0608	0.0615	0.1026	0.0635	0.1049	0.1129	0.0845	0.1173	0.1096
wk460	0.1181	0.1419	0.0822	0.0655	0.0974	0.1325	0.0679	0.0493	0.0903	0.1265	0.0563	0.1420	0.1371	0.1118	0.1733	0.1333	0.1795	0.1346	0.1364	0.0438	0.1136	0.0833	0.0859	0.1056	0.1667	0.1382
wk461	0.1582	0.1852	0.1250	0.0714	0.1369	0.1722	0.0909	0.0577	0.1118	0.1389	0.0862	0.1534	0.1694	0.1446	0.2012	0.1667	0.1928	0.1463	0.1522	0.0632	0.1600	0.1044	0.1164	0.1538	0.2143	0.1867
wk462	0.1364	0.1709	0.1090	0.0618	0.1220	0.1477	0.0640	0.0658	0.1014	0.1420	0.0471	0.1512	0.1583	0.1111	0.1813	0.1456	0.2037	0.1375	0.1418	0.0471	0.1408	0.0843	0.0870	0.1250	0.1910	0.1605
wk463	0.1313	0.1646	0.1111	0.0924	0.1059	0.1484	0.0843	0.0886	0.0974	0.1374	0.0852	0.1348	0.1613	0.1190	0.1807	0.1463	0.1845	0.1205	0.1214	0.0682	0.1267	0.0870	0.0959	0.1392	0.1902	0.1667
wk464	0.1304	0.1620	0.1000	0.0802	0.0946	0.1500	0.0833	0.0786	0.0986	0.1250	0.0759	0.1329	0.1724	0.1233	0.1875	0.1408	0.1645	0.1200	0.1190	0.0633	0.1032	0.0864	0.0902	0.1176	0.1852	0.1575
wk465	0.1538	0.1835	0.1218	0.0843	0.1341	0.1591	0.0814	0.0658	0.1133	0.1477	0.0588	0.1686	0.1552	0.1235	0.1938	0.1582	0.2160	0.1563	0.1618	0.0588	0.1690	0.0899	0.1000	0.1513	0.2079	0.1790
wk466	0.1346	0.1813	0.1203	0.1056	0.1118	0.1517	0.0747	0.0974	0.1000	0.1404	0.0640	0.1437	0.1667	0.1159	0.1747	0.1500	0.1905	0.1296	0.1357	0.0756	0.1370	0.0778	0.0845	0.1429	0.1944	0.1687
wk467	0.1420	0.1687	0.1159	0.0753	0.1279	0.1467	0.0722	0.0563	0.0962	0.1250	0.0618	0.1500	0.1532	0.1118	0.1845	0.1506	0.1882	0.1369	0.1408	0.0506	0.1600	0.0968	0.1096	0.1438	0.1935	0.1706
wk468	0.1377	0.1736	0.1071	0.0556	0.1200	0.1375	0.0833	0.0634	0.0833	0.1250	0.0714	0.1474	0.1574	0.1233	0.1736	0.1408	0.1986	0.1250	0.1356	0.0519	0.1357	0.0864	0.0942	0.1176	0.1852	0.1447
wk469	0.1286	0.1554	0.0959	0.0793	0.1104	0.1481	0.0823	0.0652	0.1000	0.1358	0.0705	0.1582	0.1532	0.1284	0.1781	0.1507	0.1974	0.1447	0.1563	0.0577	0.1172	0.0915	0.0968	0.1087	0.1768	0.1419
wk470	0.1111	0.1481	0.0864	0.0659	0.1012	0.1333	0.0682	0.0833	0.0724	0.1222	0.0682	0.1364	0.1417	0.1084	0.1646	0.1420	0.1867	0.1220	0.1214	0.0682	0.1164	0.0714	0.0694	0.1154	0.1703	0.1386
wk471	0.1136	0.1744	0.1220	0.0612	0.1111	0.0938	0.0543	0.0510	0.0889	0.1250	0.0625	0.1170	0.1290	0.0595	0.1250	0.0976	0.1591	0.1087	0.1081	0.0417	0.1842	0.0816	0.0921	0.1250	0.1939	0.1591
wk472	0.1329	0.1543	0.1063	0.0659	0.1190	0.1444	0.0625	0.0513	0.0987	0.1389	0.0690	0.1477	0.1532	0.1145	0.1829	0.1463	0.1988	0.1341	0.1304	0.0517	0.1486	0.0769	0.0833	0.1346	0.1868	0.1627
wk473	0.1081	0.1507	0.0764	0.0602	0.0987	0.1220	0.0625	0.0625	0.0833	0.1098	0.0793	0.1250	0.0962	0.0933	0.1554	0.1233	0.1467	0.0946	0.0738	0.0610	0.1154	0.0663	0.0714	0.1071	0.1627	0.1333
wk474	0.1203	0.1500	0.0949	0.0611	0.1084	0.1404	0.0747	0.0633	0.0844	0.1067	0.0747	0.1322	0.1525	0.1159	0.1728	0.1438	0.1707	0.1173	0.1087	0.0625	0.1319	0.0722	0.0775	0.1169	0.1778	0.1463
wk497	0.1125	0.1084	0.0741	0.1576	0.0882	0.0879	0.1348	0.1582	0.1104	0.1374	0.1420	0.1573	0.1148	0.0952	0.1145	0.1084	0.1726	0.1446	0.1408	0.1477	0.1081	0.1467	0.1528	0.0823	0.1087	0.0893
wk498	0.1111	0.1216	0.0811	0.1446	0.1000	0.0854	0.1188	0.1357	0.1176	0.1341	0.1203	0.1605	0.1186	0.0600	0.1026	0.0921	0.2013	0.1486	0.1462	0.1329	0.1045	0.1386	0.1508	0.0764	0.1205	0.0705
wk499	0.0962	0.1753	0.1118	0.1570	0.1076	0.1706	0.1279	0.1370	0.0903	0.1163	0.1506	0.1294	0.1727	0.1603	0.1938	0.1711	0.1375	0.0909	0.0615	0.1506	0.1176	0.1512	0.1739	0.1438	0.1977	0.1603
wk500	0.1076	0.1358	0.0813	0.0604	0.0952	0.1222	0.0511	0.0625	0.0833	0.1167	0.0511	0.1307	0.1250	0.0904	0.1585	0.1235	0.1807	0.1159	0.1159	0.0449	0.1233	0.0549	0.0563	0.1090	0.1648	0.1386
wk501	0.1429	0.1667	0.1056	0.0854	0.1000	0.1605	0.0823	0.0833	0.1164	0.1358	0.0813	0.1329	0.1864	0.1419	0.1986	0.1597	0.1645	0.1250	0.1250	0.0802	0.1172	0.0793	0.0806	0.1377	0.1951	0.1689
wk502	0.1358	0.1627	0.1037	0.0806	0.1163	0.1413	0.0667	0.0688	0.0962	0.1304	0.0506	0.1500	0.1532	0.1059	0.1726	0.1446	0.1941	0.1369	0.1408	0.0562	0.1467	0.0699	0.0753	0.1313	0.1882	0.1588
wk006	0.0641	0.1563	0.1063	0.1722	0.1190	0.1685	0.1207	0.1688	0.1053	0.1180	0.1686	0.1437	0.1855	0.1768	0.1707	0.1750	0.1488	0.1280	0.1071	0.1686	0.1370	0.1667	0.1571	0.1346	0.1833	0.1506
wk475	0.1313	0.1605	0.1000	0.0659	0.1131	0.1389	0.0511	0.0513	0.0987	0.1389	0.0517	0.1477	0.1393	0.1084	0.1707	0.1463	0.1988	0.1341	0.1357	0.0517	0.1438	0.0769	0.0833	0.1346	0.1868	0.1566
wk022	0.1415	0.1979	0.0851	0.0862	0.0943	0.1491	0.0727	0.0862	0.1019	0.1316	0.0877	0.1364	0.1406	0.1200	0.1863	0.1327	0.1635	0.1182	0.0889	0.0877	0.1200	0.0862	0.1000	0.1556	0.2155	0.1765
wk245	0.1623	0.1899	0.1346	0.0955	0.1280	0.1761	0.1047	0.0987	0.1284	0.1591	0.0941	0.1570	0.1864	0.1420	0.2063	0.1750	0.2160	0.1438	0.1493	0.0824	0.1486	0.1124	0.1232	0.1711	0.2135	0.1905
wk546	0.1056	0.1389	0.0915	0.0671	0.1067	0.1296	0.0506	0.0652	0.0597	0.1173	0.0513	0.1358	0.1569	0.0946	0.1712	0.1319	0.1908	0.1164	0.1148	0.0449	0.1328	0.0671	0.0746	0.1159	0.1707	0.1419
tm006	0.1392	0.1707	0.1111	0.0989	0.1071	0.1500	0.0795	0.0769	0.0987	0.1333	0.0747	0.1364	0.1613	0.1205	0.1768	0.1524	0.1807	0.1280	0.1304	0.0747	0.1370	0.0659	0.0704	0.1456	0.1978	0.1687
wk391	0.1284	0.1605	0.1118	0.0756	0.1076	0.1529	0.0964	0.0822	0.0959	0.1279	0.0976	0.1325	0.1754	0.1282	0.1883	0.1558	0.1795	0.1169	0.1103	0.0671	0.1232	0.0988	0.1119	0.1419	0.1977	0.1731
wk238	0.0890	0.0724	0.0063	0.1294	0.0301	0.1176	0.0976	0.1458	0.0929	0.1071	0.1111	0.1280	0.1186	0.1104	0.1188	0.1133	0.1500	0.1118	0.0923	0.1235	0.0208	0.1176	0.1364	0.0338	0.1176	0.0750
wk080	0.1173	0.1506	0.0915	0.0806	0.1047	0.1304	0.0722	0.0625	0.0897	0.1033	0.0843	0.1278	0.1210	0.1059	0.1607	0.1325	0.1647	0.1131	0.1127	0.0730	0.1200	0.0914	0.0959	0.1188	0.1720	0.1412
wk073	0.1176	0.1620	0.0845	0.0750	0.0822	0.1456	0.0649	0.0764	0.1103	0.1203	0.0769	0.1234	0.1471	0.1149	0.1620	0.1389	0.1667	0.0986	0.1017	0.0649	0.0873	0.0938	0.1111	0.1214	0.1875	0.1458
wk009	0.1250	0.1667	0.1026	0.0739	0.1173	0.1437	0.0706	0.0600	0.1014	0.1264	0.0595	0.1471	0.1466	0.1063	0.1709	0.1346	0.1938	0.1329	0.1364	0.0476	0.1338	0.0739	0.0735	0.1200	0.1875	0.1500
wk503	0.1500	0.1807	0.1235	0.0815	0.1337	0.1484	0.0787	0.0570	0.1039	0.1319	0.0739	0.1573	0.1557	0.1250	0.1807	0.1524	0.1964	0.1446	0.1500	0.0682	0.1622	0.1033	0.1164	0.1456	0.2011	0.1667
wk171	0.1370	0.1620	0.1000	0.1728	0.0987	0.1111	0.1410	0.1812	0.1418	0.1500	0.1429	0.1538	0.1455	0.1096	0.1081	0.1127	0.1892	0.1603	0.1615	0.1623	0.1014	0.1605	0.1475	0.1071	0.1543	0.1067
tm082	0.0923	0.1736	0.1212	0.2095	0.1357	0.1918	0.1408	0.2239	0.1406	0.1419	0.1929	0.1597	0.1939	0.1970	0.1791	0.2045	0.1500	0.1364	0.1230	0.2071	0.1607	0.1892	0.1638	0.1250	0.1824	0.1618
wk020	0.0775	0.1164	0.0845	0.1280	0.0800	0.1358	0.1076	0.1159	0.0809	0.0854	0.1282	0.0854	0.1186	0.1149	0.1575	0.1233	0.1118	0.0753	0.0726	0.1218	0.1016	0.1280	0.1452	0.0942	0.1341	0.1351
tm086	0.0952	0.1429	0.1389	0.1023	0.1316	0.0814	0.1023	0.1098	0.0610	0.1023	0.1512	0.0930	0.0926	0.0946	0.1750	0.0857	0.1447	0.0854	0.0588	0.1163	0.1667	0.0909	0.0833	0.1324	0.1818	0.1757
wk126	0.1139	0.1481	0.0875	0.0722	0.1024	0.1292	0.0632	0.0584	0.0800	0.0955	0.0698	0.1250	0.1290	0.1037	0.1605	0.1341	0.1646	0.1111	0.1087	0.0640	0.1111	0.0722	0.0714	0.1154	0.1722	0.1341
tm198	0.1216	0.1712	0.1111	0.0663	0.1250	0.1341	0.0783	0.0643	0.1014	0.1341	0.0759	0.1500	0.1731	0.1250	0.1835	0.1370	0.2000	0.1351	0.1452	0.0506	0.1538	0.0904	0.0870	0.1286	0.1988	0.1600
wk118	0.1282	0.1582	0.1000	0.0843	0.1159	0.1420	0.0756	0.0705	0.0987	0.1136	0.0805	0.1395	0.1525	0.1049	0.1750	0.1456	0.1728	0.1250	0.1269	0.0690	0.1458	0.0899	0.1014	0.1316	0.1854	0.1605
wk137	0.1218	0.1625	0.1013	0.0944	0.1205	0.1180	0.0632	0.0844	0.1000	0.1348	0.0756	0.1379	0.1610	0.1084	0.1747	0.1313	0.1890	0.1235	0.1304	0.0756	0.1389	0.0889	0.0959	0.1234	0.1833	0.1524
tm150	0.0786	0.0811	0.0333	0.1282	0.0329	0.1234	0.1067	0.1462	0.0794	0.0974	0.1133	0.1000	0.1389	0.1143	0.1338	0.1103	0.1357	0.1067	0.0806	0.1267	0.0441	0.1090	0.1102	0.0429	0.1410	0.0890
wk050	0.1181	0.1456	0.1026	0.0714	0.1203	0.1446	0.0556	0.0493	0.1000	0.1205	0.0688	0.1420	0.1466	0.1184	0.1667	0.1486	0.1908	0.1267	0.1129	0.0563	0.1288	0.0714	0.0781	0.1216	0.1845	0.1429
wk246	0.1250	0.1446	0.0988	0.0761	0.1105	0.1264	0.0562	0.0696	0.0909	0.1319	0.0568	0.1461	0.1393	0.0952	0.1627	0.1341	0.1964	0.1325	0.1357	0.0568	0.1419	0.0815	0.0890	0.1203	0.1793	0.1488
tm041	0.1410	0.1063	0.0949	0.2000	0.1205	0.1404	0.1724	0.2078	0.1600	0.1742	0.1802	0.1897	0.1610	0.1607	0.1543	0.1524	0.2169	0.1790	0.1884	0.1860	0.1319	0.1889	0.1831	0.1313	0.1667	0.1446
tm077	0.1014	0.1689	0.1096	0.1488	0.0974	0.1446	0.1420	0.1338	0.0870	0.0964	0.1563	0.0802	0.1228	0.1316	0.1800	0.1351	0.0974	0.1076	0.0833	0.1500	0.1284	0.1548	0.1739	0.1250	0.1548	0.1494
tm078	0.0923	0.0357	0.0347	0.1429	0.0571	0.1104	0.1149	0.1484	0.0806	0.1053	0.1301	0.1216	0.0932	0.0870	0.1250	0.0896	0.1571	0.1103	0.1091	0.1370	0.0538	0.1299	0.1579	0.0448	0.0909	0.0890
tm140	0.0903	0.1232	0.0942	0.1139	0.1042	0.1154	0.0949	0.1029	0.0758	0.0897	0.1118	0.1053	0.1000	0.0634	0.1301	0.1087	0.1458	0.0929	0.0877	0.1053	0.1311	0.1139	0.1111	0.1045	0.1203	0.1319
wk252	0.1203	0.1049	0.0938	0.1593	0.1012	0.1278	0.1193	0.1474	0.1184	0.1444	0.1379	0.1591	0.1148	0.1084	0.1280	0.1173	0.1905	0.1463	0.1377	0.1494	0.1216	0.1484	0.1644	0.1266	0.1319	0.1369
wk292	0.0441	0.0970	0.0682	0.1250	0.0652	0.1133	0.1067	0.1032	0.0556	0.0921	0.1250	0.0890	0.1132	0.1029	0.1304	0.1136	0.0942	0.0857	0.0678	0.1181	0.1048	0.1250	0.1441	0.0952	0.1382	0.1103
wk295		0.1338	0.0704	0.1358	0.0811	0.1250	0.1049	0.1408	0.0652	0.0875	0.1218	0.0949	0.1176	0.1096	0.1400	0.1181	0.1301	0.0933	0.0703	0.1282	0.1061	0.1235	0.1308	0.1029	0.1543	0.1164
wk301	0.0492		0.0584	0.1627	0.0886	0.1463	0.1375	0.1786	0.1304	0.1386	0.1519	0.1625	0.1140	0.1400	0.1486	0.1486	0.1733	0.1486	0.1385	0.1582	0.0769	0.1446	0.1719	0.0753	0.1325	0.1250
wk172	0.0480	0.0342		0.1037	0.0190	0.1037	0.0759	0.1159	0.0672	0.0864	0.0886	0.1076	0.0984	0.0878	0.1200	0.0903	0.1333	0.0890	0.0667	0.1013	0.0072	0.0854	0.0968	0.0270	0.1098	0.0714
tm005	0.0554	0.0659	0.0666		0.1163	0.1522	0.0889	0.0625	0.0962	0.1304	0.0787	0.1500	0.1452	0.1294	0.1845	0.1506	0.1941	0.1369	0.1408	0.0562	0.1400	0.0860	0.0890	0.1313	0.1882	0.1588
wk180	0.0448	0.0362	0.0282	0.0643		0.1118	0.0904	0.1301	0.0775	0.0941	0.1037	0.0964	0.1271	0.1090	0.1235	0.1053	0.1188	0.0844	0.0606	0.1159	0.0143	0.1047	0.1194	0.0400	0.1221	0.0802
wk242	0.0420	0.0448	0.0420	0.0645	0.0411		0.1180	0.1456	0.1169	0.1429	0.1307	0.1629	0.1129	0.0714	0.1205	0.0610	0.2024	0.1506	0.1500	0.1420	0.1267	0.1413	0.1181	0.1013	0.1413	0.1131
wk390	0.0530	0.0680	0.0662	0.0362	0.0642	0.0595		0.0714	0.0933	0.1180	0.0523	0.1264	0.1356	0.0793	0.1488	0.1188	0.1768	0.1111	0.1029	0.0698	0.1111	0.0778	0.0822	0.1104	0.1667	0.1402
wk068	0.0551	0.0663	0.0629	0.0376	0.0618	0.0589	0.0346		0.1067	0.1266	0.0823	0.1494	0.1122	0.1111	0.1831	0.1357	0.1597	0.1127	0.1034	0.0506	0.1694	0.0813	0.0952	0.1418	0.1875	0.1667
tm032	0.0352	0.0536	0.0534	0.0531	0.0498	0.0508	0.0503	0.0526		0.0705	0.1104	0.0933	0.1200	0.1071	0.1522	0.1176	0.1197	0.0764	0.0583	0.0974	0.0833	0.0897	0.0862	0.1000	0.1474	0.1143
wk505	0.0340	0.0505	0.0498	0.0510	0.0476	0.0498	0.0509	0.0538	0.0262		0.1307	0.0899	0.1148	0.1250	0.1687	0.1280	0.1250	0.0843	0.0775	0.1250	0.1014	0.1304	0.1458	0.1013	0.1467	0.1310
wk165	0.0524	0.0643	0.0644	0.0356	0.0618	0.0584	0.0332	0.0363	0.0490	0.0501		0.1453	0.1293	0.0864	0.1625	0.1329	0.1975	0.1313	0.1343	0.0625	0.1268	0.0674	0.0725	0.1184	0.1798	0.1420
wk028	0.0347	0.0538	0.0526	0.0526	0.0496	0.0515	0.0519	0.0535	0.0276	0.0284	0.0494		0.1417	0.1463	0.1852	0.1481	0.1250	0.0864	0.0797	0.1453	0.1042	0.1444	0.1571	0.1234	0.1667	0.1524
tm098	0.0491	0.0446	0.0454	0.0656	0.0428	0.0428	0.0655	0.0674	0.0538	0.0521	0.0640	0.0529		0.0556	0.1636	0.0556	0.1667	0.1429	0.1556	0.1466	0.1226	0.1371	0.1630	0.0648	0.0565	0.1404
wk030	0.0425	0.0459	0.0437	0.0614	0.0404	0.0329	0.0609	0.0615	0.0493	0.0477	0.0591	0.0493	0.0371		0.1299	0.0732	0.1818	0.1316	0.1364	0.0988	0.1269	0.1235	0.1364	0.1013	0.1059	0.1039
wk309	0.0422	0.0428	0.0417	0.0609	0.0400	0.0321	0.0624	0.0635	0.0498	0.0473	0.0600	0.0492	0.0364	0.0272		0.1351	0.2179	0.1733	0.1719	0.1813	0.1250	0.1726	0.1739	0.1164	0.1548	0.1203
wk311	0.0401	0.0428	0.0425	0.0606	0.0399	0.0298	0.0595	0.0621	0.0491	0.0474	0.0576	0.0496	0.0372	0.0259	0.0261		0.1800	0.1351	0.1439	0.1329	0.1000	0.1506	0.1508	0.1104	0.1145	0.1200
tm020	0.0365	0.0537	0.0526	0.0538	0.0489	0.0510	0.0524	0.0545	0.0313	0.0321	0.0511	0.0311	0.0523	0.0481	0.0478	0.0486		0.0779	0.0448	0.1914	0.1471	0.1941	0.2121	0.1301	0.1529	0.1772
tm019	0.0388	0.0556	0.0537	0.0523	0.0492	0.0472	0.0476	0.0495	0.0355	0.0350	0.0471	0.0360	0.0545	0.0515	0.0496	0.0486	0.0342		0.0634	0.1313	0.1111	0.1310	0.1328	0.0986	0.1488	0.1316
tm161	0.0352	0.0501	0.0492	0.0526	0.0465	0.0498	0.0521	0.0530	0.0318	0.0290	0.0497	0.0311	0.0523	0.0484	0.0473	0.0481	0.0327	0.0357		0.1343	0.0932	0.1338	0.1509	0.1148	0.1549	0.1406
tm096	0.0537	0.0663	0.0644	0.0292	0.0628	0.0606	0.0325	0.0348	0.0504	0.0501	0.0332	0.0516	0.0645	0.0598	0.0611	0.0590	0.0515	0.0481	0.0518		0.1479	0.0730	0.0797	0.1250	0.1854	0.1605
tm145	0.0489	0.0365	0.0329	0.0660	0.0238	0.0491	0.0685	0.0684	0.0547	0.0502	0.0673	0.0529	0.0418	0.0428	0.0423	0.0428	0.0518	0.0558	0.0507	0.0670		0.1333	0.1525	0.0391	0.1333	0.0903
wk495	0.0527	0.0637	0.0630	0.0353	0.0607	0.0602	0.0352	0.0362	0.0523	0.0495	0.0336	0.0518	0.0632	0.0584	0.0584	0.0572	0.0524	0.0486	0.0517	0.0341	0.0653		0.0342	0.1188	0.1774	0.1471
wk496	0.0565	0.0646	0.0643	0.0372	0.0643	0.0654	0.0398	0.0419	0.0574	0.0519	0.0401	0.0553	0.0667	0.0619	0.0608	0.0607	0.0571	0.0534	0.0535	0.0391	0.0663	0.0189		0.1148	0.1986	0.1515
wk211	0.0412	0.0420	0.0406	0.0594	0.0379	0.0328	0.0577	0.0593	0.0470	0.0458	0.0566	0.0469	0.0343	0.0313	0.0308	0.0300	0.0467	0.0459	0.0455	0.0571	0.0415	0.0570	0.0605		0.0813	0.0461
wk212	0.0423	0.0399	0.0388	0.0589	0.0387	0.0330	0.0600	0.0595	0.0506	0.0464	0.0590	0.0500	0.0351	0.0316	0.0300	0.0306	0.0488	0.0505	0.0464	0.0597	0.0415	0.0581	0.0597	0.0271		0.1118
wk215	0.0411	0.0437	0.0416	0.0611	0.0389	0.0311	0.0572	0.0595	0.0491	0.0478	0.0550	0.0493	0.0376	0.0337	0.0339	0.0312	0.0476	0.0465	0.0477	0.0567	0.0441	0.0566	0.0618	0.0259	0.0290	
